# Off-the-shelf CAR natural killer cells secreting IL-15 target spike in treating COVID-19

**DOI:** 10.1038/s41467-022-30216-8

**Published:** 2022-05-11

**Authors:** Ting Lu, Rui Ma, Wenjuan Dong, Kun-Yu Teng, Daniel S. Kollath, Zhiyao Li, Jinhee Yi, Christian Bustillos, Shoubao Ma, Lei Tian, Anthony G. Mansour, Zhenlong Li, Erik W. Settles, Jianying Zhang, Paul S. Keim, Bridget M. Barker, Michael A. Caligiuri, Jianhua Yu

**Affiliations:** 1 Department of Hematology and Hematopoietic Cell Transplantation, City of Hope National Medical Center, Los Angeles, CA 91010 USA; 2 Hematologic Malignancies Research Institute, City of Hope National Medical Center, Los Angeles, CA 91010 USA; 3 Pathogen and Microbiome Institute, Northern Arizona University, Flagstaff, AZ 86011 USA; 4 Department of Computational and Quantitative Medicine, City of Hope National Medical Center, Los Angeles, CA 91010 USA; 5 Division of Pathogen and Microbiome, TGen North, Flagstaff, AZ 86011 USA; 6 City of Hope Comprehensive Cancer Center, Los Angeles, CA 91010 USA; 7 Department of Immuno-Oncology, Beckman Research Institute of City of Hope, Los Angeles, CA 91010 USA

**Keywords:** Viral infection, Immunotherapy, Lymphocyte activation, SARS-CoV-2

## Abstract

Engineered natural killer (NK) cells represent a promising option for immune therapy option due to their immediate availability in allogeneic settings. Severe acute diseases, such as COVID-19, require targeted and immediate intervention. Here we show engineering of NK cells to express (1) soluble interleukin-15 (sIL15) for enhancing their survival and (2) a chimeric antigen receptor (CAR) consisting of an extracellular domain of ACE2, targeting the spike protein of SARS-CoV-2. These CAR NK cells (mACE2-CAR_sIL15 NK cells) bind to VSV-SARS-CoV-2 chimeric viral particles as well as the recombinant SARS-CoV-2 spike protein subunit S1 leading to enhanced NK cell production of TNF-α and IFN-γ and increased in vitro and in vivo cytotoxicity against cells expressing the spike protein. Administration of mACE2-CAR_sIL15 NK cells maintains body weight, reduces viral load, and prolongs survival of transgenic mice expressing human ACE2 upon infection with live SARS-CoV-2. These experiments, and the capacity of mACE2-CAR_sIL15 NK cells to retain their activity following cryopreservation, demonstrate their potential as an allogeneic off-the-shelf therapy for COVID-19 patients who are faced with limited treatment options.

## Abstract (web summary)

Severe COVID-19 requires immediate and targeted intervention that is efficient against SARS-CoV-2 and its variants. Authors show here the therapeutic potential of engineered natural killer cells that simultaneously express a chimeric antigen receptor targeting the spike protein of SARS-CoV-2, and IL-15, a cytokine that enhances the function and survival of their own.

## Introduction

The outbreak of coronavirus disease of 2019 (COVID-19), caused by the novel severe acute respiratory syndrome coronavirus-2 (SARS-CoV-2), has escalated into a pandemic^1^. As of March 2022, >472 million COVID-19 cases were reported globally and there were more than 6.1 million deaths^2^. Not surprisingly, the public health and economic consequences have been devastating. Currently, the standard of care for COVID-19 patients includes oxygen therapy and ventilation along with the antiviral remdesivir and the anti-inflammatory dexamethasone. Remdesivir^3,4^ and dexamethasone^5^ have been approved for emergency therapeutic use for COVID-19, and each agent improved patient outcomes in clinical trials. However, remdesivir has limited efficacy^6^ and dexamethasone is a steroid without direct antiviral efficacy. Recently, the first oral antiviral drug for COVID-19, Lagevrio, was approved by the Medicines and Healthcare products Regulatory Agency in the UK to treat mild-to-moderate COVID-19^7^. Later, Paxlovid was approved by the US FDA for emergency use to treat mild-to-moderate COVID-19 disease in adults and pediatric patients over 12 years of age^7^. To date, the FDA has also approved four therapeutic monoclonal antibodies for emergency use to treat mild-to-moderate COVID-19 patients: imdevimab, casirivimab, bamlanivimab, and etesevimab^7^. Cellular immunotherapies for COVID-19 patients have not been approved yet, but they could be beneficial because they harness existing immunity to fight the disease.

Natural killer (NK) cells are innate immune lymphocytes that recognize and rapidly lyse abnormal cells, including virally infected cells, allogeneic cells, and tumor cells, without antigen pre-sensitization or human leukocyte antigen (HLA) matching^8,9^. Although NK cells are universal killers in the immune response against certain viruses or tumors, genetically modifying them to express chimeric antigen receptors (CAR) can further improve their targeting abilities^10^. Thus, NK cell lines, primary NK cells from peripheral blood and umbilical cord blood (UCB), and induced pluripotent stem cells have been used to manufacture CAR NK cells^11^. Like peripheral blood, UCB is a rich source of primary human NK cells and a readily available donor source with known HLA genotyping and specific NK receptor profiles; ~800,000 UCB units have been stored in public cord blood banks and >5,000,000 in private cord blood banks^12^. Recently, a clinical trial that incorporated IL-15 into UCB CAR NK cells targeting the CD19 antigen showed impressive outcomes in treating certain lymphoid malignancies^13^. These results provide an encouraging foundation for the clinical use of CAR NK products.

Due to the rapid and potent nature of innate immune responses to viral infection, NK cells should be capable of fighting COVID-19, but endogenous NK cells lack specificity against SARS-CoV-2. However, the surface of SARS-CoV-2 is covered with the glycosylated spike (S) protein that can bind to the host cell receptor angiotensin-converting enzyme 2 (ACE2), mediating viral entry^14^. The S protein consists of an N-terminal subunit (S1), which mediates receptor binding, and a C-terminal subunit (S2) responsible for fusing the virus to the cell membrane^15^. Neutralizing antibodies that target the receptor-binding domain of S1 can inhibit infection by blocking binding to ACE2^16–20^. Although NK cells do not naturally express the receptor ACE2 on their surface, we speculated that engineering NK cells to express ACE2 should enable them to target SARS-CoV-2-infected cells.

In this study, we isolate NK cells from UCB and generate CAR NK cells using a mutant (m) extracellular domain of ACE2 along with human soluble IL-15 (sIL15). We refer to these cells as mACE2-CAR_sIL15 NK cells. First, we test the ability of cells to bind the SARS-CoV-2 spike protein and their cytotoxic efficacy against cells expressing the spike protein. We also determine whether infusing mACE2-CAR_sIL15 NK cells into transgenic mice expressing human ACE2 can target the S protein and protect against infection by live SARS-CoV-2 and COVID-19-like illness in vivo. Our frozen off-the-shelf allogeneic mACE2-CAR_sIL15 NK cells show robust efficacy against cells expressing the spike protein in vitro and against live SARS-CoV-2 infection in vivo. Our product provides a novel immunotherapeutic approach for treating COVID-19 infection and other SARS infections expressing the spike protein.

## Results

### Generation of mACE2-CAR_sIL15 NK cells

**Fig. 1:**
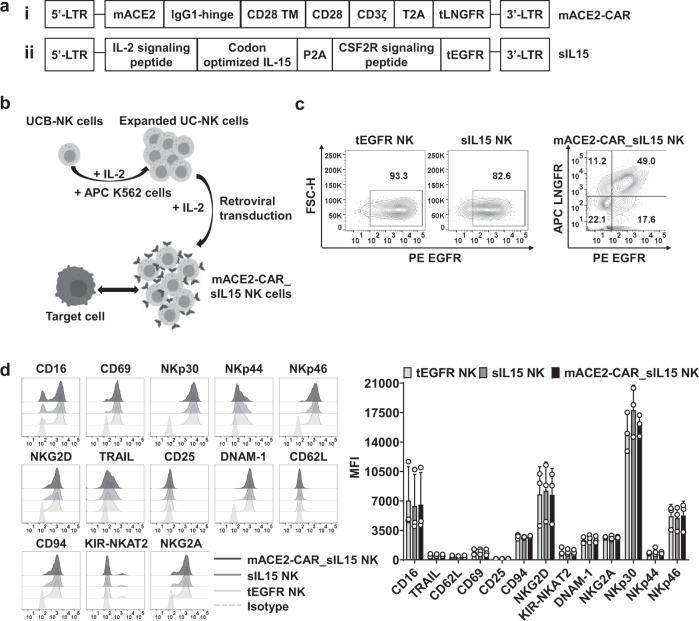
Generation of mACE2-CAR_sIL15 NK cells. **a** Design of the mACE2-CAR and sIL15 retroviral vectors. The mACE2-CAR vector contains a mutant human ACE2, a human IgG1-hinge region, and the CD28 transmembrane region, followed by the intracellular domains of CD28 and CD3ζ linked to a T2A and a truncated (t) LNGFR (i). The sIL15 vector contains an IL-2 signaling peptide, a codon-optimized IL-15, and the GM-CSF receptor (CSF2R) signaling peptide, followed by a truncated (t) EGFR (ii). **b** Generation of mACE2-CAR_sIL15 NK cells. The pictures were created with BioRender.com. **c** The transduction efficiency of control tEGFR or sIL15 NK cells and mACE2-CAR_sIL15 NK cells was determined by flow cytometry. Control tEGFR or sIL15 NK cells expressed the tEGFR marker, while mACE2-CAR_sIL15 NK cells expressed the tLNGFR maker (representing mACE2-CAR expression) as well as the tEGFR marker (representing sIL15 expression). **d** The phenotypic analysis of mACE2-CAR_sIL15 NK cells and control tEGFR or sIL15 NK cells were determined by flow cytometry. Representative flow cytometry histograms and summary data from three different donors are shown. *P* values were determined by one-way ANOVA with multiple comparisons and adjusted by the Holm-Sidak method. Data are presented as mean ± SD. For mACE2-CAR_sIL15 NK cells, sIL15 expression is denoted as LNGFR^–^EGFR^+^, mACE2-CAR expression is denoted as LNGFR^+^EGFR^–^, and sIL15 & mACE2-CAR_co-expression is denoted as LNGFR^+^EGFR^+^. Source data are provided as a Source Data file.

To develop CAR NK cell-based immunotherapy to treat SARS-CoV-2 infection, we combined a mutant fragment of ACE2 (mACE2)—the zinc metallopeptidase domain^21^—to a CD28 and CD3ζ intracellular signaling domain to create mACE2-CAR (Fig. 1a). We also isolated NK cells from UCB and cultured them for 5 days with antigen-presenting K562 feeder cells expressing both 4-1BBL and membrane-bound (mb) IL-21 (APC K562). To generate mACE2-CAR_sIL15 NK cells, we transduced the expanded and activated NK cells with a mixture of two retroviral vectors. One expressed mACE2-CAR and truncated (t) low-affinity nerve growth factor receptor (LNGFR); the other expressed sIL15 and truncated (t) epidermal growth factor receptor (EGFR) (Fig. 1a). Following transduction, the UCB NK cells were cultured for an additional 10 days (Fig. 1b), the transduction efficiency of control tEGFR NK cells or control sIL15 NK cells was evaluated by measuring tEGFR expression. CAR expression on mACE2-CAR_sIL15 NK cells was determined by measuring tLNGFR expression, while sIL15 expression on mACE2-CAR_sIL15 NK cells was evaluated by measuring tEGFR expression (gating strategy in Supplementary Fig. 1a). About half of the NK cells co-expressed mACE2-CAR and sIL15 (Fig. 1c and Supplementary Fig. 1b, c). When we evaluated the stability of mACE2-CAR_sIL15 expression in our CAR NK cells, we found that over 90% of FACS-sorted or purified mACE2-CAR_sIL15 NK cells co-expressed mACE2-CAR and sIL15 after culture in vitro for 14 days (Supplementary Fig. 1d, e). To determine whether IL-15 could activate the NK cells, we comprehensively characterized the mACE2-CAR_sIL15 NK cell phenotype, including for expression of activating and inhibitory receptors on expanded NK cell products by flow cytometry. Our data show that the phenotype of mACE2-CAR_sIL15 NK cells resembles that of control NK cells transduced with only tEGFR or of NK cells transduced with sIL15 (Supplementary Fig. 1f and Fig. 1d), likely because all three types of NK cells were activated and expanded in identical fashion by the APC K562 in the presence of IL-2. This result is consistent with a recent report describing prostate stem-cell antigen (PSCA) CAR NK cells co-expressing sIL15^22^.

### mACE2-CAR_sIL15 NK cells bind to SARS-CoV-2 spike protein and VSV-SARS-CoV-2 chimeric viral particles

**Fig. 2:**
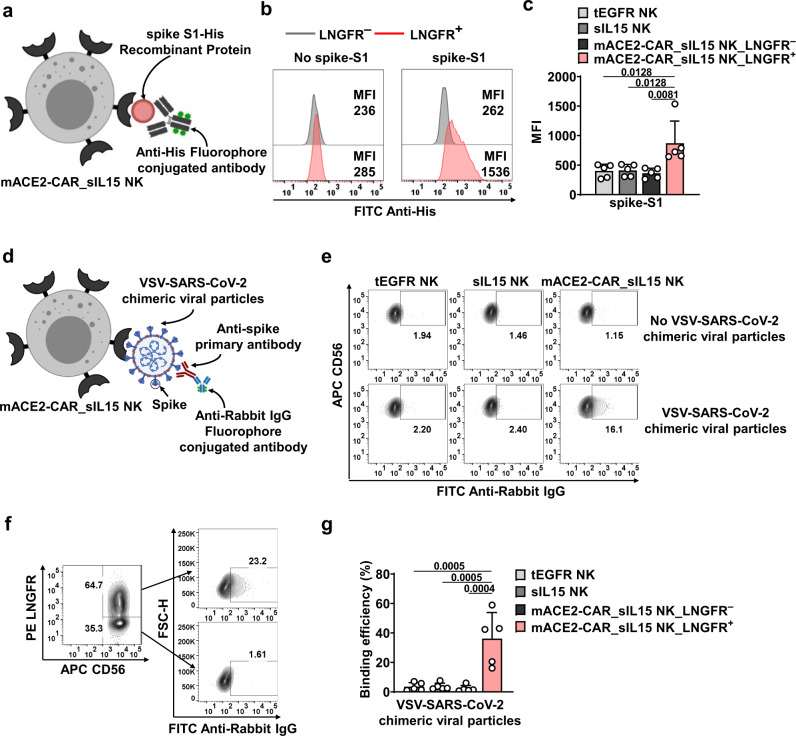
mACE2-CAR_sIL15 NK cells bind to SARS-CoV-2 spike protein and VSV-SARS-CoV-2 chimeric viral particles. **a** Diagram of mACE2-CAR_sIL15 NK cells binding to spike S1-His-tagged recombinant protein. MFI = mean fluorescence intensity. The picture was created with BioRender.com. **b**, **c** Representative flow cytometry plots showing the results of mACE2-CAR_sIL15 NK cells binding to spike S1-His-tagged recombinant protein (**b**). LNGFR was used as a marker for mACE2-CAR-positive NK cells. Summary data from five different donors are shown in **c**. *P* values were determined by one-way ANOVA with multiple comparisons and adjusted by the Holm–Sidak method. Data are presented as mean ± SD. **d** Diagram of spike S1-His-tagged recombinant protein binding to VSV-SARS-CoV-2 chimeric viral particles, which are recognized by anti-spike and its corresponding fluor-conjugated secondary antibody. The picture was created with BioRender.com. **e** Representative flow cytometry plots showing the efficiency of control NK or mACE2-CAR_sIL15 NK cells binding to VSV-SARS-CoV-2 chimeric viral particles. **f**, **g** Representative flow cytometry plots showing the efficiency of CAR-negative or CAR-positive subsets in mACE2-CAR_sIL15 NK cells binding to VSV-SARS-CoV-2 chimeric viral particles. Summary data from five different donors are shown in **g**. *P* values were determined by one-way ANOVA with multiple comparisons and adjusted by the Holm-Sidak method. Data are presented as mean ± SD. Source data are provided as a Source Data file.

After generating the mACE2-CAR_sIL15 NK cells, we wanted to confirm their ability to bind the SARS-CoV-2 spike protein. We, therefore, incubated mACE2-CAR_sIL15 NK and control NK cells with a recombinant His-tagged spike S1 and assessed complex formation by flow cytometry, using an anti-His-conjugated antibody (Fig. 2a). The SARS-CoV-2 spike S1 protein subunit bound to the CAR-positive population of mACE2-CAR_sIL15 NK cells but not to either the CAR-negative population or control NK cells transduced with tEGFR or sIL15 (Fig. 2b, c and Supplementary Fig. 2a). Because the SARS-CoV-2 spike S1 protein subunit alone may not fully reflect the complexity of SARS-CoV-2 viral particles, we next determined whether mACE2-CAR_sIL15 NK cells could bind to viral particles expressing the SARS-CoV-2 spike protein. Thus, we incubated mACE2-CAR_sIL15 NK and control NK cells with chimeric vesicular stomatitis virus (VSV) particles expressing the SARS-CoV-2 spike protein (VSV-SARS-CoV-2)^23^. We then assessed complex formation by flow cytometry, using an anti-spike protein antibody and its corresponding fluor-conjugated secondary antibody (Fig. 2d). As expected, mACE2-CAR_sIL15 NK cells bound to the VSV-SARS-CoV-2 viral particles, whereas neither control NK cells transduced with tEGFR or sIL15 nor the CAR-negative population of mACE2-CAR_sIL15-transduced NK cells was able to bind (Fig. 2e–g). We, therefore, concluded that mACE2-CAR_sIL15 NK cells can specifically bind to the SARS-CoV-2 spike S1 protein subunit and to viral particles expressing the SARS-CoV-2 spike protein.

### mACE2-CAR_sIL15 enhances NK cell cytotoxicity against target cells expressing SARS-CoV-2 spike protein

**Fig. 3:**
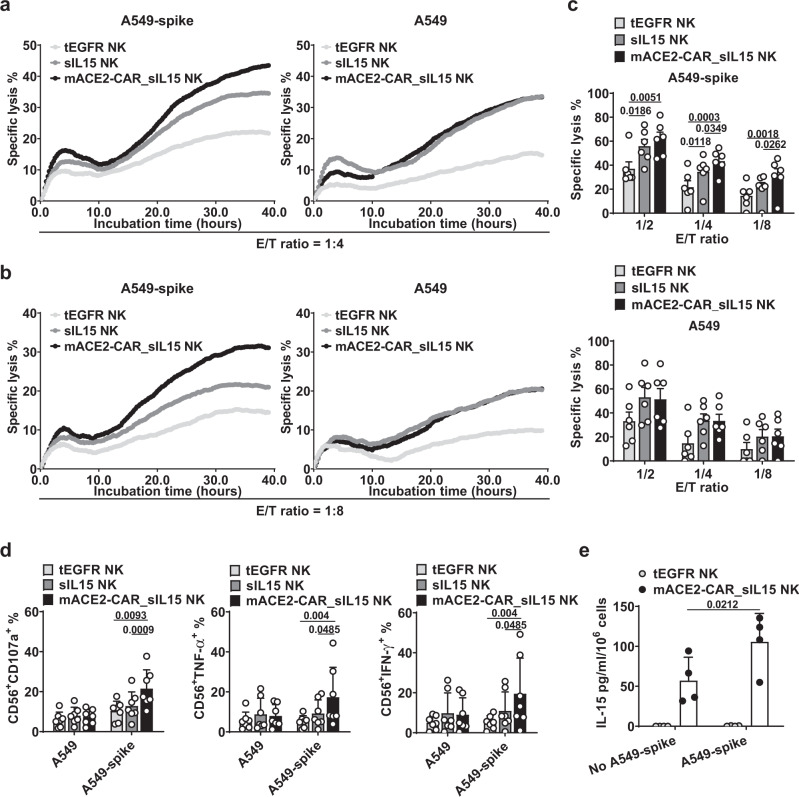
mACE2-CAR_sIL15 enhances NK cell cytotoxicity against target cells that express SARS-CoV-2 spike protein. **a** Representative real-time cell analysis (RTCA) data showing NK cell cytotoxicity against A549-spike or parental A549 cells at an effector (E)/target (T) ratio of 1:4. **b** RTCA data showing NK cell cytotoxicity against A549-spike or parental A549 cells at an E/T ratio of 1:8. **c** Data from the RTCA assay analyzed at three different E/T ratios are summarized for cells from 6 donors. *P* values were determined with one-way ANOVA with multiple comparisons and adjusted by the Hochberg method. Data are presented as mean ± SEM. **d** Expression of CD107a, TNF-α, and IFN-γ was measured on transduced NK cells co-cultured with A549-spike or parental A549 cells for 4 h at an E/T ratio of 4:1. Data are summarized for NK cells from 7 donors. *P* values were determined by one-way ANOVA with multiple comparisons and adjusted by the Holm-Sidak or Dunn’s method. Data are presented as mean ± SD. **e** IL-15 production by ex vivo-expanded control tEGFR NK cells or mACE2-CAR_sIL15 NK cells cultured in the presence or absence of A549-spike cells for 72 h. Data are summarized for NK cells from four donors. *P* values were determined by one-way ANOVA with multiple comparisons and adjusted by the Holm–Sidak method. Data are presented as mean ± SD. Source data are provided as a Source Data file.

To determine whether mACE2-CAR_sIL15 NK cells display enhanced effector function after interaction with target cells that express the SARS-CoV-2 spike, we expressed the spike protein in a human lung carcinoma cell line, A549 (A549-spike). We next incubated mACE2-CAR_sIL15 NK and control NK cells with A549-spike or parental A549 cells and assessed the effector cells’ ability to eradicate A549-spike or parental A549 cells. Using a long-term cytotoxicity assay, real-time cell analysis (RTCA), we found that mACE2-CAR_sIL15 NK cells significantly exhibited greater cytotoxicity against A549-spike cells than control NK cells (Fig. 3a, b, and Supplementary Fig. 2b). Summarizing cytotoxic activity at different effector/target (E/T) ratios showed that mACE2-CAR_sIL15 NK cells were significantly more cytotoxic against A549-spike cells compared to control NK cells. However, a similar increase did not occur when parental A549 cells served as target cells (Fig. 3c). Next, we measured levels of NK cell degranulation, as well as TNF-α and IFN-γ production, as parameters of NK cell function. Consistent with our cytotoxicity results, mACE2-CAR_sIL15 NK cells significantly increased CD107a degranulation and the secretion of TNF-α and IFN-γ in the presence of A549-spike cells compared to tEGFR or sIL15 control NK cells. However, there was no difference between the mACE2-CAR_sIL15 NK and control NK cells in the presence of parental A549 cells (Fig. 3d and Supplementary Fig. 2c). We also measured IL-15 release after culturing mACE2-CAR_sIL15 NK cells and control tEGFR NK cells in the presence or absence of A549-spike cells for 72 h. IL-15 was undetectable in supernatants from control tEGFR NK cells. On the other hand, mACE2-CAR_sIL15 NK cells produced small amounts of IL-15 (mean 57.2 pg/ml/10^6^ cells, range 31.6–94.3 pg/ml/10^6^ cells) in the absence of A549-spike cells and significantly more (mean 105.6 pg/ml/10^6^ cells, range 54.9–135.4 pg/ml/10^6^ cells) when cultured with A549-spike cells (Fig. 3e). Collectively, these results indicate that mACE2-CAR_sIL15 NK cells exhibit specific and robust NK cell effector function in the presence of target cells that express the SARS-CoV-2 spike protein, mimicking a response to cells infected with SARS-CoV-2.

### Freeze-thawed mACE2-CAR_sIL15 NK cells show effective anti-spike activity in vitro and in vivo

**Fig. 4:**
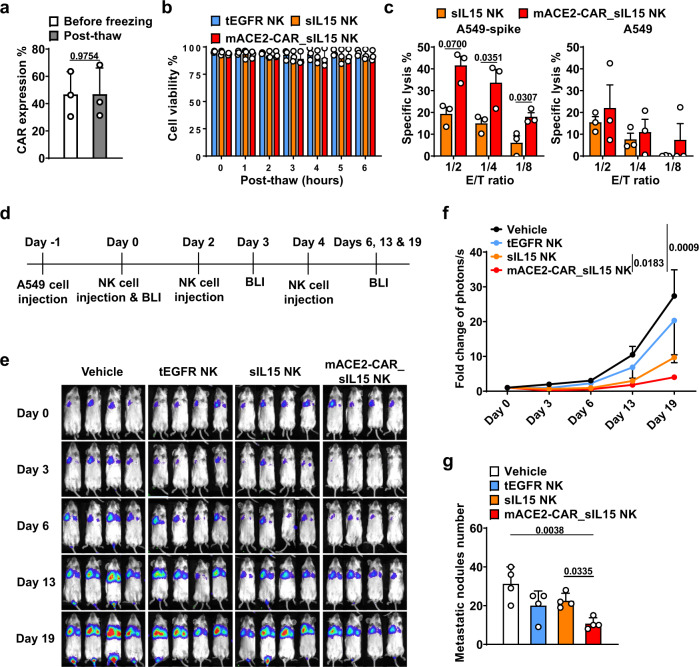
Freeze-thawed mACE2-CAR_sIL15 NK cells show effective anti-spike activity in vitro and in vivo. **a** CAR expression in mACE2-CAR_sIL15 NK cells post-thaw, as determined by flow cytometry. Data are summarized for cells from three donors. *P* value was determined by two-tailed paired *t* test. Data are presented as mean ± SD. **b** Cell viability of mACE2-CAR_sIL15 NK cells as determined at the indicated time points post-thaw using the Muse Cell Analyzer. Data are summarized for NK cells from three donors. *P* values were determined by two-way ANOVA with repeated measures and adjusted by the Holm-Sidak method. Data are presented as mean ± SD. **c** Lysis of freeze-thawed control NK or mACE2-CAR_sIL15 NK cells that were co-cultured with A549-spike or parental A549 cells and analyzed with real-time cell analysis (RTCA). Data are summarized for NK cells from three donors. *P* values were determined with a two-tailed paired *t* test. Data are presented as mean ± SEM. **d** Scheme for in vivo studies using NSG mice. **e** Tumor growth in NSG mice inoculated with firefly luciferase-labeled A549-spike cells, as monitored by changes in tumor bioluminescence. Colors indicate intensity of luminescence. **f** Summary of tumor burden data from **e** (four mice/group). *P* values were determined by one-way ANOVA with with multiple comparisons and adjusted by the Holm-Sidak method on day 19. Data are presented as mean ± SEM. **g** After hematoxylin and eosin (H&E) staining of lung tissues, tumor burden was identified and evaluated as the number of tumor nodules, which is defined as ≥50 µm. Four fields of Regions of Interest (ROIs) per lung were selected, and the number of lung tumor nodules per four fields was counted. Summary data are shown (four mice/group). *P* values were determined by one-way ANOVA with multiple comparisons and adjusted by the Holm-Sidak method. Data are presented as mean ± SD. Source data are provided as a Source Data file.

To understand the potential clinical applicability of mACE2-CAR_sIL15 NK cells, we evaluated their stability after cryopreservation in vitro right after a freeze-thaw cycle. More than 80% of the cells showed functional recovery, and cell viability was over 90% (Supplementary Fig. 3a, b). Moreover, mACE2-CAR expression in the mACE2-CAR_sIL15 NK cells post-thaw was similar to that of fresh mACE2-CAR_sIL15 NK cells (Fig. 4a). Moreover, mACE2-CAR_sIL15 NK cell viability persisted at >80%, even at 6 h post-thaw (Fig. 4b). Next, we evaluated the potency of cryopreserved mACE2-CAR_sIL15 NK cells. The thawed mACE2-CAR_sIL15 NK cells were more cytotoxic against A549-spike cells but not against parental A549 cells when compared to thawed control sIL15 NK cells (Fig. 4c).

We then used a firefly luciferase (FFLuc)-labeled A549-spike xenograft NOD-SCID-IL2Rγ^–/–^ (NSG) mouse model to study the in vivo anti-spike activity of mACE2-CAR_sIL15 NK cells. The NSG mice (8–12 weeks old) were inoculated intravenously (i.v.) with FFLuc-labeled A549-spike cells on day −1. On each of days 0, 2, and 4, the mice received an i.v. infusion (10 × 10^6^/mouse) of the vehicle (PBS) only, freeze-thawed control tEGFR NK cells, control sIL15 NK cells, or mACE2-CAR_sIL15 NK cells (Fig. 4d). Tumor growth was monitored by measuring changes in tumor bioluminescence over time (Fig. 4e). Infusion of mACE2-CAR_sIL15 NK cells improved tumor control compared to treatment with the vehicle or control sIL15 NK cells (Fig. 4f). On day 20, the mice were euthanized, and tissues were harvested and prepared for detection of human NK cells; they were also formalin-fixed and paraffin-embedded (FFPE). Using flow cytometry (Supplementary Fig. 4a), we identified human NK cells in mouse blood, spleen, and liver (Supplementary Fig. 4b) and detected the expression of mACE2-CAR on those cells (Supplementary Fig. 4c), indicating the survival and successful homing of mACE2-CAR_sIL15 NK cells 20 days post-infusion. Moreover, the presence of A549-spike cells in the lung was confirmed with H&E staining (Supplementary Fig. 4d). The number of lung metastases was significantly lower in mice treated with mACE2-CAR_sIL15 NK cells than in those treated with control sIL15 NK cells (Fig. 4g). Taken together, our in vitro and in vivo data show that mACE2-CAR_sIL15 NK cells retain cytotoxic activity after freezing. We next investigated whether mACE2-CAR_sIL15 NK cells have potential application for the treatment of infection caused by SARS-CoV-2 or its variants or any virus that uses ACE2 to enter cells.

### mACE2-CAR_sIL15 NK cells protect against live SARS-CoV-2 infection in the K18-hACE2 transgenic mouse model

**Fig. 5:**
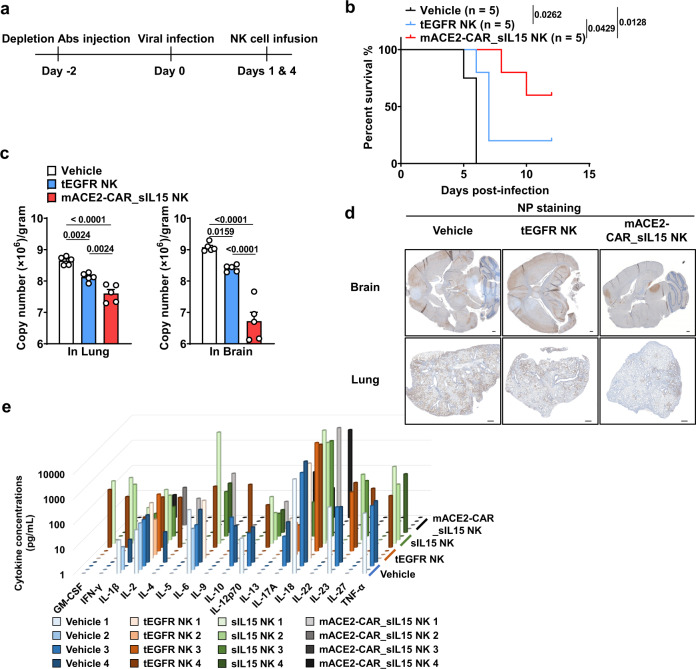
mACE2-CAR_sIL15 NK cells protect against live SARS-CoV-2 infection in the K18-hACE2 humanized mouse model. **a** Scheme for in vivo studies using K18-hACE2 humanized transgenic mice. **b** K18-hACE2 humanized transgenic mice were infected with 1 × 10^3^ plaque-forming units (PFU) of SARS-CoV-2 prior to being treated with the vehicle (PBS), control tEGFR NK cells, or mACE2-CAR_sIL15 NK cells. Survival of mice is summarized. *N* = 5 mice/group. *P* values were determined by Kaplan–Meier survival analysis and calculated by the Gehan-Breslow-Wilcoxon test (two-sided). **c** Viral RNA levels are shown for brain and lung tissues of mice infected i.n. with 1 × 10^3^ plaque-forming units (PFU) of SARS-CoV-2. *N* = 5 mice/group. *P* values were determined by one-way ANOVA with multiple comparisons and adjusted by the Holm-Sidak method. Data are presented as mean ± SEM. **d** Representative images of the three groups in **b** show immunohistochemistry (IHC) staining of coronavirus nucleocapsid protein (NP). Scale bars, 400 μm. **e** Release assay of various cytokines into plasma of K18-hACE2 transgenic mice. Mice were infected with 1 × 10^3^ PFU of live SARS-CoV-2 prior to being treated with the vehicle (PBS), control tEGFR NK cells, control sIL15 NK cells, or mACE2-CAR_sIL15 NK cells. Four days later, all mice were sacrificed to collect blood plasma to measure levels of the indicated cytokines by a cytokine release Luminex assay. *N* = 4 mice/group. Data are presented as an average of two replicates for each mouse. Source data are provided as a Source Data file.

To determine whether mACE2-CAR_sIL15 NK cells can prevent infection with live SARS-CoV-2 in vivo, we used K18-hACE2 transgenic mice, which express human ACE2 in epithelial airway cells^24^. Following infection with live SARS-CoV-2, untreated K18-hACE2 transgenic mice began to lose weight on days 2 through 4 and to die of the inflammatory response on days 6 through 9^25,26^. This response was consistent with our recent characterization of K18-hACE2 mice after live SARS-CoV-2 infection^27^. In our experiment, two days before intranasal (i.n.) infection on day 0 with live SARS-CoV-2, these mice were depleted of endogenous immune cells to avoid potential rejection of human NK cells. On day 1, the mice were treated with the vehicle (PBS) only, control NK cells, or mACE2-CAR_sIL15 NK cells (Fig. 5a). Body weight was monitored daily after infection and calculated as a percentage of initial body weight. When the mice were infected with 1 × 10^2^ plaque-forming units (PFU) of live SARS-CoV-2, the control tEGFR NK cells protected them from the infection, as body weight dropped significantly less than that of the vehicle-treated mice (Supplementary Fig. 5a). However, mice that received mACE2-CAR_sIL15 NK cells after 1 × 10^2^ PFU of live SARS-CoV-2 infection maintained their body weight significantly better than those that received control tEGFR NK cells (Supplementary Fig. 5a). When infection was undertaken with a higher dose (1 × 10^3^ PFU) of live SARS-CoV-2, mice receiving mACE2-CAR_sIL15 NK cells again had the best protection from SARS-CoV-2 infection as measured by maintenance of body weight (Supplementary Fig. 5b). All the vehicle-treated mice died prior to day 6, and most mice treated with control tEGFR or sIL15 NK cells died prior to day 7 (Fig. 5b and supplementary Fig. 5c). However, all mice treated with mACE2-CAR_sIL15 NK cells were surviving on day 6, and three of the five mice (60%) treated with mACE2-CAR_sIL15 NK cells recovered and survived for at least 12 days (Fig. 5b). Similar data were obtained with a longer observation time of 24 days (Supplementary Fig. 5c).

To further investigate protection against SARS-CoV-2 infection by mACE2-CAR_sIL15 NK cells, we used quantitative reverse transcriptase (RT)-PCR to measure viral spike protein RNA levels in the brain and lung of mice infected with 1 × 10^3^ PFU of live SARS-CoV-2. The mice that received mACE2-CAR_sIL15 NK cells had significantly lower viral copies of SARS-CoV-2 in brain and lung tissues compared to the mice that received the vehicle or control tEGFR NK cells (Fig. 5c); they also had lower expression of coronavirus nucleocapsid protein (NP) (Fig. 5d). These data with live SARS-CoV-2 further support our conclusion that mACE2-CAR-sIL15 NK cells can limit SARS-CoV-2 infection.

To evaluate the safety profile of our CAR NK cells, we measured levels of 17 cytokines in plasma of mice infected with 1 × 10^3^ PFU of live SARS-CoV-2 and treated with the vehicle, control tEGFR NK cells, control sIL15 NK cells, or mACE2-CAR_sIL15 NK cells. Levels of those cytokines, which included IL-6, IL-10, IL-17A, IFN-γ, TNF-α, and GM-CSF, did not differ significantly between the mACE2-CAR_sIL15 NK cell group and any of the three control groups (Fig. 5e and Supplementary Fig. 5d). These preclinical data suggest that treatment with mACE2-CAR_sIL15 NK cells may not induce substantial cytokine toxicity when used to treat COVID-19 patients.

## Discussion

This study supports the concept that engineered NK cells can be an effective therapy against SARS-CoV-2 infection. Because NK cells do not express the ACE2 target protein for SARS-CoV-2, we utilized UCB to generate CAR NK cells that overexpress an extracellular domain of ACE2. These engineered NK cells bound to recombinant SARS-CoV-2 spike protein and VSV-SARS-CoV-2 chimeric viral particles. As mature NK cells can survive only briefly in vivo in both humans and mice^25^, we also transduced CAR NK cells with a gene encoding human sIL15, a crucial cytokine for NK cell persistence and activation^26–30^. NK cells with sIL15 expression, but not those without sIL15 expression, were detectable in mice 20 days post-infusion, supporting IL-15’s importance in maintaining this cell product in vivo. Because the large size of an all-in-one vector might limit transduction efficiency, we used a two-vector approach, which increased efficiency but required each vector to be manufactured separately with a relatively higher cost. In vitro and in vivo, our mACE2-CAR_sIL15 NK cells showed striking efficacy compared to control NK cells, in cytotoxic activity, in reduction of SARS-CoV-2 viral copies, and in reduction of SARS-CoV-2-associated death. The potential clinical benefit of this cellular therapeutic approach is supported by a recent clinical trial that utilized partially HLA matched or HLA unmatched allogeneic UCB to generate anti-CD19 CAR NK cells along with IL-15 to produce significant anti-tumor activity against CD19-positive lymphoid malignancies^13^. The results of our study are also supported by some of our previous work, which demonstrated that NK cells can clear herpes simplex virus (HSV) 1 infection in vivo because of the receptor complex that recognizes a target expressed by HSV^31^. The availability of a frozen, off-the-shelf, allogeneic CAR NK cell-based therapy for high-risk COVID-19 patients without other approved treatment options, could have immediate clinical benefits. Finally, the fact that our cryopreserved product’s potency resembles that of our fresh product suggests that allogeneic mACE2-CAR_sIL15 NK cells could be cryopreserved, shipped, and then stored at any medical center for immediate thawing and infusion at the first sign of clinical deterioration in any high-risk individual infected with SARS-CoV-2.

The immunopathology of SARS-CoV-2 is based on the dysfunction of both the innate and antigen-specific immune responses. Many studies have revealed that COVID-19 patients have significantly decreased numbers of NK cells and cytotoxic T cells compared to non-infected controls^32–34^, and patients with severe COVID-19 have fewer of these cytolytic cells than patients with mild COVID-19^35^. Furthermore, the NK cell inhibitory receptor NKG2A^32^ and T-cell exhaustion markers PD-1 and Tim-3^34^ are overexpressed in COVID-19 patients as compared to healthy donors. Upon encountering virus-infected cells, mACE2-CAR_sIL15 NK cells can robustly secrete cytokines such as IFN-γ and TNF-α. Also, the soluble IL-15 that they produce maintains human mACE2-CAR_sIL15 NK cell survival in vivo and enhances the antiviral activity of endogenous immune cells such as CD8^+^ T cells and NK cells. Moreover, IL-15 is known to be essential for T cell homeostasis^36,37^. In human monocytes, IL-15 can induce the production of IL-18 and monocyte chemotactic protein 1, which attracts neutrophils and monocytes to sites of infection^38^. In macrophages, IL-15 also functions as a potent autocrine regulator of proinflammatory cytokine production^39^. Taken together, these attributes suggest that the addition of an immune effector cell therapy, such as mACE2-CAR_sIL15 NK cells, may correct, at least in part, immunological deficiency caused by SARS-CoV-2 infection.

CAR NK cells may also offer several advantages over CAR T cells for COVID-19 patients. Although both CAR NK cell and CAR T cell therapies use engineered immune cells to recognize and kill cells expressing a specific antigen, there are important differences. CAR T cells, the first CAR-engineered cellular immunotherapy that has been approved by the FDA, have a long history of clinical use. However, allogeneic T cells can induce graft-versus-host disease, whereas allogeneic NK cells do not^40,41^, the latter of which opens a door for the broader therapeutic application of NK cells^42^. Also, allogeneic CAR NK cells appear less likely than autologous CAR T cells to cause cytokine release syndrome (CRS), a potentially fatal complication due to the release of IL-6, IFN-γ, IL-1, etc.^13,43^. Limiting CRS is especially important for COVID-19 patients, who often display a cytokine storm even in the absence of such therapies, with increased levels of inflammatory cytokines and chemokines (TNF-α, IL-1, IL-6, IL-8, IL-10, IL-18, and MCP-1) that severely damage pulmonary tissue^34,44–46^. Our in vivo study showed that levels of numerous cytokines, including IL-6, IL-10, IL-17A, IFN-γ, TNF-α, and GM-CSF, were similar in the mACE2-CAR_sIL15 NK cell group and either the vehicle, control tEGFR NK cell group, or control sIL15 NK cell group. Further, unlike their T-cell counterparts, NK cells do not recall antigens following multiple exposures, thereby limiting the possibility of serious toxicity as has been seen in most CAR T-cell studies. Thus, our frozen, allogeneic off-the-shelf mACE2-CAR_sIL15 NK cell product is unlikely to induce a toxic cytokine storm when given future consideration for the treatment of COVID-19 patients.

The COVID-19 pandemic is a global health crisis and with the worldwide emergency of current beta and omicron variants, there is no indication that it will end soon. In the current study, we generated a novel, ready-to-use, and off-the-shelf frozen allogeneic cellular product for treating COVID-19. Unlike recently reported CAR NK cells, generated from peripheral blood NK cells and transduced with an scFv domain of an anti-spike antibody^47^, our CAR NK cell product was based on the ACE2 receptor itself, which exists in humans as a natural cellular entry point for the SARS-CoV-2 virus. We were able to test our product in the K18-hACE2 transgenic mouse model showing reduced survival following infection with live SARS-CoV-2 virus. Our product can therefore also be considered for the treatment of other coronaviruses that use the spike protein to penetrate host cells, such as the SARS-CoV-1 that spawned the epidemic of 2003^48^. The worldwide abundance of public UCB banks provides a rich source of donor NK cells that can then be expanded, transduced, and frozen for immediate shipping or storage for future use. Indeed, growing and cryopreserving a rich source of the UCB-derived mACE2-CAR_sIL15 NK cell product for future, more virulent SARS-CoV epidemics would seem prudent. Following Good Manufacturing Practice and FDA clearance, the mACE2-CAR_sIL15 NK cell product could be tested in a clinical trial for moderate to high-risk patients infected with SARS-CoV-2, especially for those who may not require intensive care or mechanical ventilation, as happened in a recent clinical trial using activated, non-engineered NK cells to treat COVID-19 patients^49^. The outcome of that trial suggests that an NK cell-based approach is generally safe and may be effective for such patients. Our strategy of adding the specificity of a mACE2-CAR to NK cells and the expression of sIL15 may improve efficacy with less frequent dosing thereby limiting toxicity.

While this study provides evidence for a potential therapeutic pathway for the treatment of infections by SARS-CoV-2 or other viruses that express the spike protein, the approach may have several limitations. For instance, we do not yet know if our promising in vivo results will translate into success in clinical trials. Unlike cancer patients who get lymphodepleting chemotherapy^13^, COVID-19 patients may not be suitable for lymphodepletion due to the ongoing infection. However, our allogeneic mACE2-CAR_sIL15 NK cells express IL-15, which can sustain NK cell survival in treated patients infected by SARS-COV-2, as demonstrated in cancer patients^13^. NK cells have numerous inhibitory receptors, which can help to protect the CAR NK cells from being rejected by the recipient. Also, overexpressing HLA-E and knocking out β2m in our CAR NK cells may reduce rejection by host NK cells and T cells, respectively, as currently demonstrated for universal CAR T cells^50–52^. However, we also do not know if allogeneic off-the-shelf mACE2-CAR_sIL15 NK cells could produce intolerable adverse events in COVID-19 patients or simply prove ineffective in humans. Clinical trials with high-risk COVID-19 patients will be required to determine if the product is safe, well-tolerated, and effective.

In summary, we have generated a novel immunotherapeutic approach for treating SARS-CoV-2 infection with frozen, off-the-shelf allogeneic mACE2-CAR_sIL15 NK cells. Our human CAR NK cell product proved efficacious in reducing morbidity and mortality of COVID-19 as caused by live SARS-CoV-2 virus infecting a transgenic mouse model expressing hACE2.

## Methods

### Ethics statement

Experiments and handling of mice were conducted under federal, state, and local guidelines and with approval from the City of Hope Animal Care and Use Committee and Northern Arizona University. Human NK cells were isolated from UCB cells after written informed consent under a protocol approved by the City of Hope Institutional Review Board.

### Study design

This study was designed to create and test a novel NK cell product for eliminating SARS-CoV-2-from infected cells and potentially treating COVID-19 in humans. UCB NK cells or those derived from UCB hematopoietic stem cells were engineered to express a CAR with the extracellular domain of the SARS-CoV-2 target protein ACE2, providing specificity for SARS-CoV-2 infection, and IL-15. The NK cells expressing the CAR and IL-15 were tested for their ability to target SARS-CoV-2 and kill SARS-CoV-2-infected cells both in vitro and in vivo.

### Cell lines

The A549 cell line was purchased from the American Type Culture Collection and cultured in Roswell Park Memorial Institute (RPMI) with 10% heat-inactivated FBS (Sigma-Aldrich). The APC K562 cell line was previously described^53^ and cultured in RPMI with 10% heat-inactivated FBS (Sigma-Aldrich). The GP2-293 packaging cell line was purchased from Takara Bio and cultured in DMEM supplemented with 1% GlutaMax and 10% FBS. All cells were incubated at 37°C in a 5% CO_2_ humidified incubator. No further authentication of these cell lines was performed after a recent purchase. Cell morphology and growth characteristics were monitored during the study and compared with published reports to ensure their authenticity. All cell lines were routinely tested for the absence of mycoplasma using the MycoAlert Mycoplasma Detection Kit from Lonza.

### Plasmid construction and retrovirus production

The retroviral vector encoding tEGFR, sIL15, and mACE2-CAR were constructed after multiple steps of PCR amplification, gel electrophoresis and extraction, enzyme digestion, ligation, transformation, and plasmid extraction.

To generate retroviral particles, the GP2-293 cells were cultured to a confluency of 70–80% and then transfected with the constructed retroviral vectors with the envelope plasmid RD114TR by using the Lipofectamine 3000 Reagent (ThermoFisher Scientific). The culture supernatant containing the retrovirus was harvested at 48 h post-transfection and filtered.

### Generation of CAR-modified NK cells

UCB units were provided from StemCyte under IRB-approved protocols. All donors provided written informed consent, which followed the ethical guidelines of the Declaration of Helsinki. NK cells were isolated by using the RosetteSep^TM^ human NK cell enrichment cocktail (Cat# 15065, StemCell Technologies) and Ficoll-Paque (Cat# 17144003, Cytiva). The purity of primary NK cells was confirmed with flow cytometry using anti-CD56 (Cat# IM2474U, Beckman Coulter; 1:20 dilution) and anti-CD3 (Cat# 130-113-134, Miltenyi Biotec; 1:50 dilution) antibodies. Frozen UCB NK cells were thawed and expanded with irradiated K562 feeder cells expressing membrane-bound IL-21 and 4-1BBL (APC K562) in the presence of recombinant human IL-2 (50 IU/ml; NIH) in Stem Cell Growth Medium (SCGM) (Cat# 20802-0500, CellGenix). Expanded NK cells were transduced with retrovirus on day 5 in RetroNectin (Cat# T202, Takara Bio)-coated plates, according to the manufacturer’s protocol. On day 8, NK cells were co-cultured with irradiated APC K562 cells for an additional 7 days prior to being harvested for in vitro analysis or frozen (liquid nitrogen) for in vitro and in vivo studies. All NK cells used in our study did not undergo further purification, except where specifically indicated. Information on flow antibodies was presented in Supplementary Table 1.

### Cytotoxicity assays

For RTCA cytotoxicity assays, A549 or A549-spike cells (A549 cells engineered to express the spike protein) were used as target cells. First, 50 µl of cell culture medium was added to each well of an E-plate (Cat# 300601010, Agilent). The E-plate is a standard 96-well plate with a glass-bottom coated with gold microelectrodes covering approximately 75% of the well area. The E-plate was then connected to the system to check for proper electrical contacts and to obtain background impedance readings in the absence of cells. Target cells (5000 cells in 100 µl of media) were plated into the E-plate and cultured overnight in the RTCA system installed in the CO_2_ incubator. mACE2-CAR_sIL15 NK and control NK cells in 100 µl media were added into the E-plate and co-cultured for at least an additional 40 h in the RTCA system. The proliferation of cytotoxicity of target cells was analyzed and plotted using the RTCA software Pro every 15 min in a real-time manner. The cytotoxicity of each effector was calculated with the following equation: % of cytolysis = (CI_no effector_ − CI_effector_)/CI_no effector_ × 100.

### CD107a degranulation and intracellular cytokine production

mACE2-CAR_sIL15 NK and control NK cells were co-cultured with A549 or A549-spike cells at an E/T ratio of 4:1 for 4 h in a 96-well U-bottom plate. Anti-CD107a monoclonal antibody (mAb) (Cat# 563869, BD; 1:200 dilution) and GolgiPlug (1:1000 dilution) (Cat# 555029, BD) were added to cultures at the start of incubation. Cells were stained with anti-CD56 (Cat# IM2474U, Beckman Coulter; 1:20 dilution), anti-LNGFR (Cat# 557196, BD; 1:20 dilution) or anti-EGFR (Cat# 352904, BioLegend; 1:50 dilution) mAbs and then stained intracellularly with IFN-γ (Cat# 563563, BD; 1:20 dilution) and TNF-α (Cat# 557647, BD; 1:20 dilution) mAbs. The stained cells were analyzed by flow cytometry, and the data were analyzed with FlowJo software.

### IL-15 cytokine secretion

Supernatants were harvested after mACE2-CAR_sIL15 NK and control NK cells were co-cultured without or with A549-spike cells for 72 h. IL-15 concentrations were measured with the human IL-15 Quantikine ELISA kit (Cat# S1500, R&D) following the manufacturer’s instructions. Each experiment was performed in triplicate.

### Assessment of binding of mACE2-CAR_sIL15 NK cells to a recombinant spike S1 protein subunit

mACE2-CAR_sIL15 NK and control NK cells (2 × 10^5^) were incubated with 2 µg of a recombinant S1 protein subunit for 2 h at 37 °C. The cells were washed twice and stained with FITC anti-His (Cat# MA1-81891, ThermoFisher; 1:20 dilution), APC anti-CD56, PE anti-LNGFR, or anti-EGFR mAbs for 20 min at room temperature. After washing, the stained cells were analyzed by flow cytometry. Data were analyzed with FlowJo software.

### Assessment of binding of mACE2-CAR_sIL15 NK cells to VSV-SARS-CoV-2 chimeric viral particles

VSV-SARS-CoV-2 chimeric viral particles were added into mACE2-CAR_sIL15 NK and control NK cells (2 × 10^5^) in a 96-well V-bottom plate. The plate was centrifuged at 600 × *g* for 30 min at 37 °C and then incubated for 1 h. The cells were washed twice and stained with an anti-S1 antibody at 37 °C for 30 min. The cells were washed twice and stained with a FITC goat anti-rabbit secondary antibody (Cat# 554020, BD; 1:20 dilution), APC anti-CD56, PE anti-LNGFR, or anti-EGFR mAbs for 20 min at room temperature. After washing, cells were analyzed by flow cytometry, and the data were analyzed with FlowJo software.

### NSG xenograft model

NSG mice (Stain# 005557) were purchased from the Jackson Laboratory and housed at the City of Hope Animal Facility. On day −1, female NSG mice (8–12 weeks old) were inoculated intravenously (i.v.) with FFLuc-labeled A549-spike cells (3.5 × 10^5^). On each of days 0, 2, and 4, they received an i.v. infusion (10 × 10^6^/mouse) of the vehicle, freeze-thawed control tEGFR NK cells, control sIL15 NK cells or mACE2-CAR_sIL15 NK cells (four mice per group; three infusions per mouse). Tumor growth was monitored by measuring changes in tumor bioluminescence over time. Bioluminescence imaging was performed with Lago-Spectral Instruments Imaging on days 0, 3, 6, 13, and 19. On day 20, mice were euthanized by CO_2_ inhalation, and tissues were harvested and prepared as FFPE blocks.

### Humanized K18-hACE2 mouse model

Heterozygous K18-hACE2 C57BL/6 J mice (2B6.Cg-Tg(K18-ACE2)2Prlmn/J) (Stain# 034860) were obtained from The Jackson Laboratory. K18-hACE2 transgenic mice (females, 6–8 weeks old) received 200 µg each of anti-NK1.1 mAb (Cat# BE0036, BioCell), anti-mCD4 (Cat# BE0003-1, BioCell), and anti-mCD8a (Cat# BP0061, BioCell) plus 100 µl clodronate liposomes (Cat# CLD-8909, Encapsula NanoSciences) for macrophage depletion via intraperitoneal (i.p.) injection on day −2. Two days later (day 0), the mice were i.n. infected with SARS-CoV-2. On day 1, the mice received i.v. administration of the vehicle, 15 × 10^6^ control tEGFR NK cells, control sIL15 NK cells, or mACE2-CAR_sIL15 NK cells. Bodyweight was monitored daily after infection and calculated as a percentage of initial body weight. Work with SARS-CoV-2 was performed in a biosafety level 3 laboratory by personnel equipped with powered air-purifying respirators.

### Detecting viral RNA copy number

The details were given in our previous study^54^. Briefly, the viral RNA was isolated from the homogenized tissues using the PureLink RNA Mini kit (Invitrogen). A one-step RT-PCR kit (BioRad) was used to detect the viral RNA, using Applied Biosystems QuantStudio 12 K Flex Real-Time PCR System. The primer sequences were CoV-2-S_19F, 5′-GCTGAACATGT-CAACAACTC-3′ and CoV-2-S_143R, 5′-GCAATGATGGATTGACTAGC-3′. The standard samples were serial 10-fold dilutions of a known copy number. The results were normalized and expressed as genome equivalent copies per gram of tissue.

### Statistical analysis

Two independent or paired groups were compared by Student’s two-tailed *t* tests or paired *t* tests. One-way or two-way ANOVA was used in multiple groups comparison with adjusted *p* values. A linear mixed model was used in repeated measures from the same subjects. Survival data were analyzed by the Kaplan–Meier method and compared by the Log-rank test or Gehan-Breslow-Wilcoxon test. A *p* value less than 0.05 was defined as statistically significant. GraphPad 9.1.0 was used for statistical analysis.

### Reporting summary

Further information on research design is available in the Nature Research Reporting Summary linked to this article.

## Supplementary information

Supplementary Information

Reporting Summary

## Data Availability

All original data generated in this study are provided in the Supplementary Information/Source Data file. Source data are provided with this paper.

## Source data

Source Data
