# Supplementary material for: Off-the-shelf CAR natural killer cells secreting IL-15 target spike in treating COVID-19
Source: Nat Commun. 2022 May 11;13:2576. doi: 10.1038/s41467-022-30216-8 (PMC9095674; doi:10.1038/s41467-022-30216-8)
Supplement: Supplementary file 1 — Supplementary Information [file 41467_2022_30216_MOESM1_ESM.pdf]

Supplementary Information

Off-the-Shelf CAR Natural Killer Cells Secreting IL-15 Target Spike in Treating COVID-19

Ting Lu[1, 2, †], Rui Ma[1†], Wenjuan Dong[1†], Kun-Yu Teng[1], Daniel S Kollath[3], Zhiyao Li[1], Jinhee Yi[3], Christian Bustillos[1], Shoubao Ma[1], Lei Tian[1], Anthony G. Mansour[1], Zhenlong Li [1], Erik W Settles[3], Jianying Zhang[4], Paul S Keim[3,5], Bridget M. Barker[3], Michael A. Caligiuri[1,2,6*], and Jianhua Yu[1,2,6,7*]

[1]Department of Hematology and Hematopoietic Cell Transplantation, City of Hope National Medical Center, Los Angeles, CA 91010, USA.

[2]Hematologic Malignancies Research Institute, City of Hope National Medical Center, Los Angeles, CA 91010, USA.

[3]Pathogen and Microbiome Institute, Northern Arizona University, Flagstaff, AZ 86011, USA.

[4]Department of Computational and Quantitative Medicine, City of Hope National Medical Center, Los Angeles, CA 91010, USA.

[5]Division of Pathogen and Microbiome, TGen North; Flagstaff, AZ 86011, USA

[6]City of Hope Comprehensive Cancer Center, Los Angeles, CA 91010, USA.

[7]Department of Immuno-Oncology, Beckman Research Institute of City of Hope, Los Angeles, CA 91010, USA.

[†]Equally contributing authors

*Corresponding authors. Email: Michael A. Caligiuri, MD, mcaligiuri@coh.org and Jianhua Yu, PhD, jiayu@coh.org.

Supplementary Fig. 1

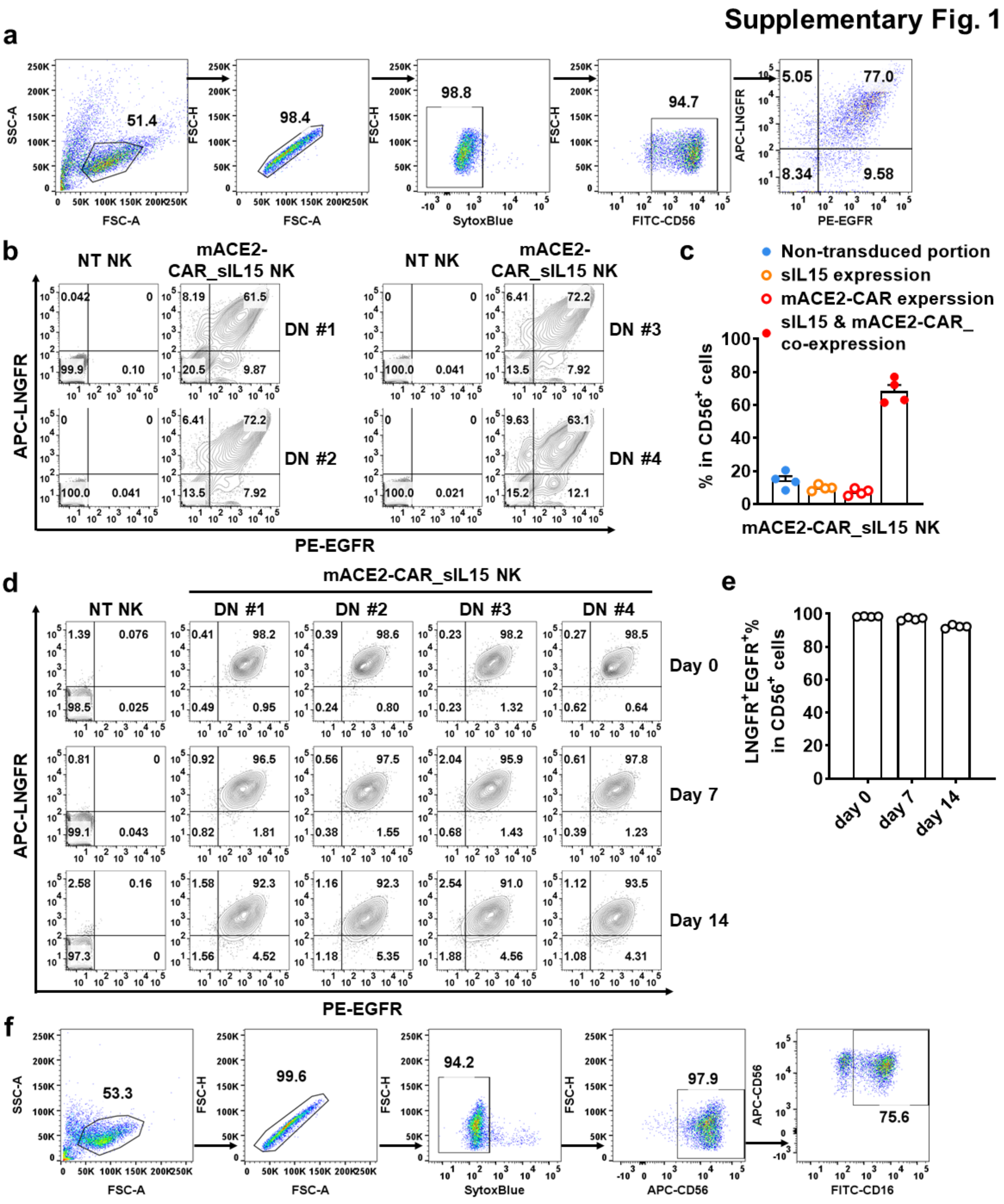

Supplementary Figure 1. (a) Gating strategy to determine the percentage of LNGFR^{+}EGFR^{+} NK cells. **(b, c)** The transduction efficiency of mACE2-CAR_sIL15 NK cells was determined by flow

cytometry, with the percentage of LNGFR representing mACE2-CAR expression and the percentage of EGFR representing sIL15 expression. Representative flow cytometry plots (**b**) and summary data (**c**) from 4 different donors are shown. **(d, e)** The mACE2-CAR and sIL15 double-positive NK cells were sorted from four different donors and then continued to be cultured for 2 weeks. Expression of mACE2-CAR and sIL15 in these cells was detected at day 7 and day 14 post culture via flow cytometry. Representative flow cytometry plots (**d**) and summary data (**e**) from 4 different donors are shown. NT, non-transduced; DN, donor. Data are presented as mean ± SD. **(f)** Gating strategy to determine the surface markers on NK cells.

Supplementary Fig. 2

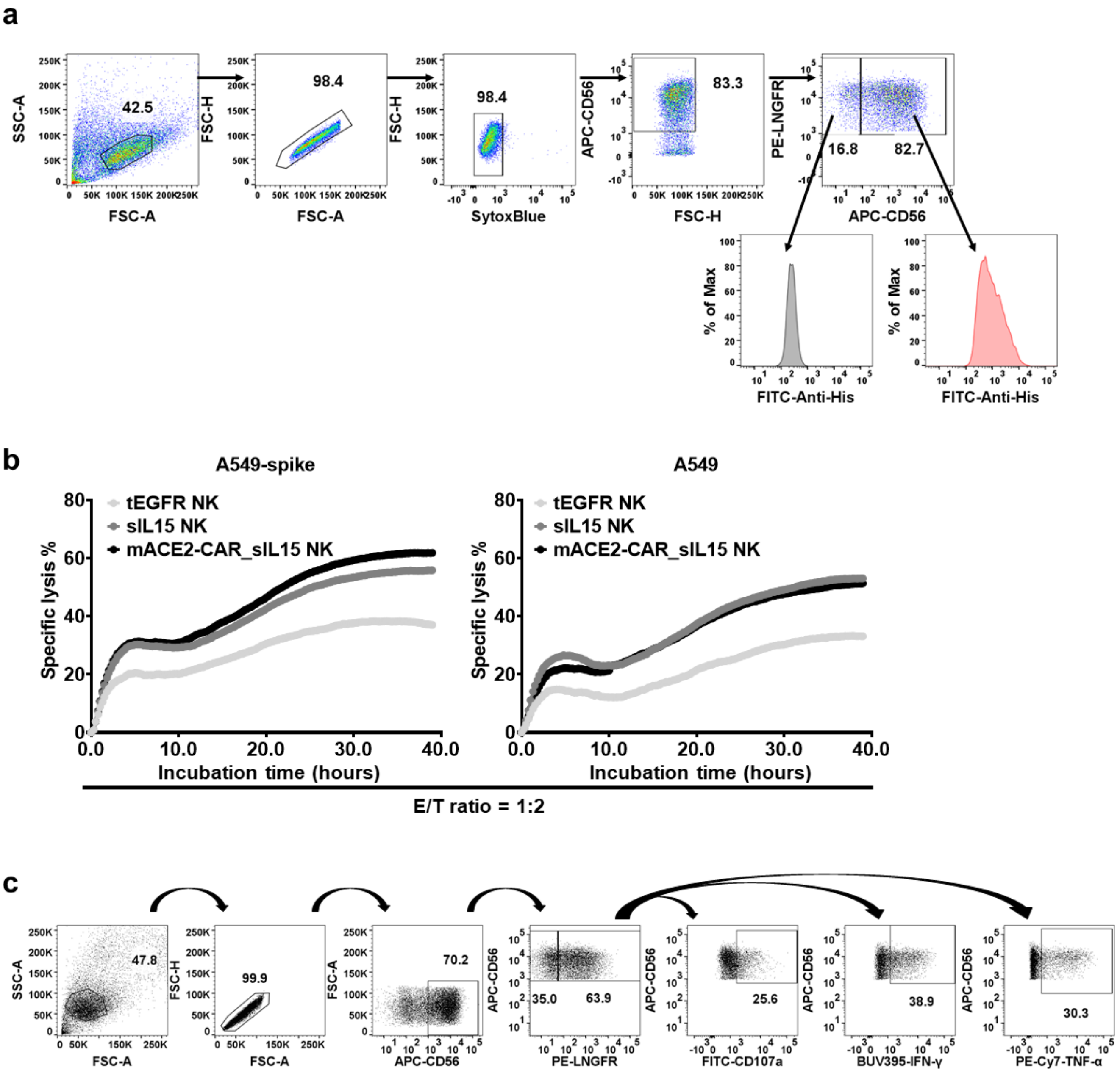

Supplementary Figure 2. (a) Gating strategy to determine the percentage of spike protein or VSV-SARS-CoV-2 chimeric viral particles binding in NK cells. **(b)** Representative real-time cell analysis (RTCA) assay data showing NK cell cytotoxicity against A549-spike or parental A549 cells at an effector/target (E/T) ratio of 1:2. **(c)** Representative flow cytometry plots showing general gating strategy for the percentages of $CD56^{+}CD107a^{+}$, $CD56+IFN\text{-}\gamma^{+}$ and $CD56+TNF\text{-}\alpha^{+}$ in transduced NK cells.

Supplementary Fig. 3

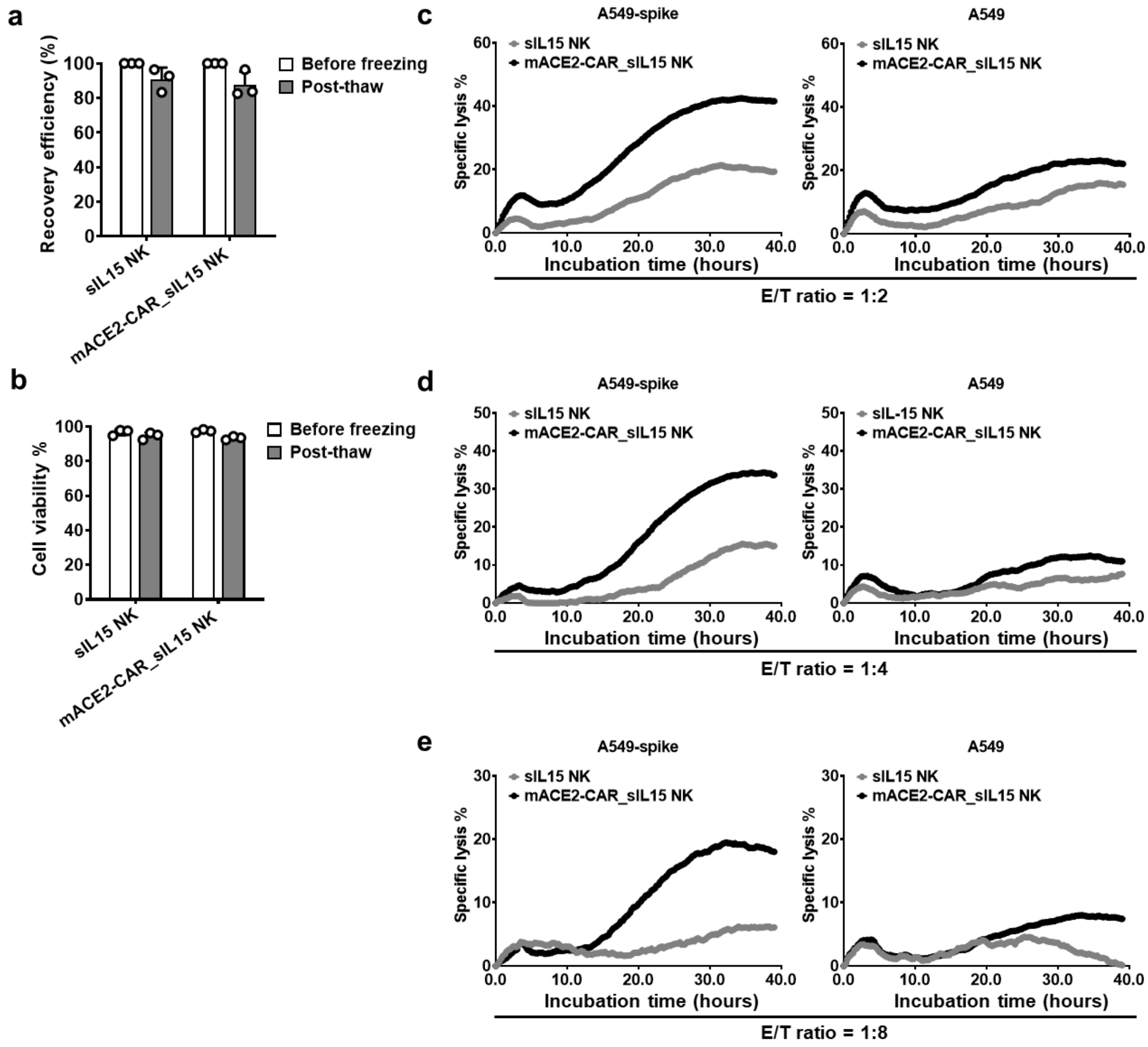

Supplementary Figure 3. (a) CAR expression in mACE2-CAR_sIL15 NK cells post-thaw, as determined by flow cytometry. Data are summarized from 3 different donors. (**b**) Cell viability of mACE2-CAR_sIL15 NK cells prior to freezing and post-thaw was determined using the Muse Cell Analyzer. Data are summarized from 3 different donors. **(c-e)** Freeze-thawed sIL15 control NK or mACE2-CAR_sIL15 NK cells were co-cultured with A549-spike or parental A549 cells for real-time cell analysis (RTCA) assay. Representative RTCA assay data showing NK cell cytotoxicity against A549-spike or parental A549 cells at effector/ target (E/T) ratios of 1:2 (**c**), 1:4 (**d**), and 1:8 (**e**). Data are presented as mean ± SD.

Supplementary Fig. 4

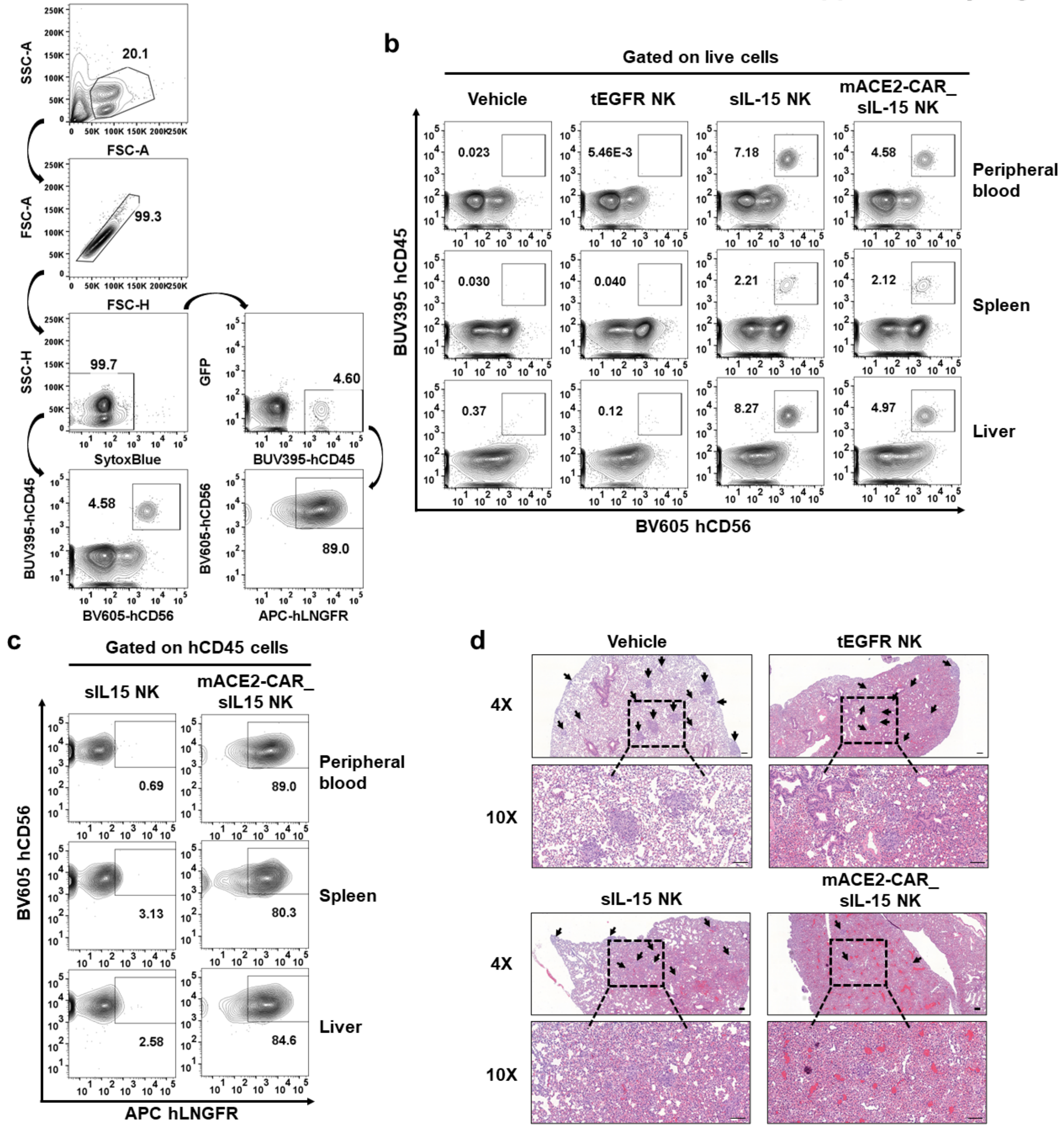

Supplementary Figure 4. (a-c) The A549-spike engrafted NSG mice were treated with PBS, control tEGFR, control sIL15, or mACE2-CAR_sIL15 NK cells (4 mice/group), followed by euthanasia on day 20. Peripheral blood, spleens, and livers were harvested and analyzed for expression of human (h)CD45 (a human lymphocyte marker) by flow cytometry. Representative flow cytometry plots showing general gating strategy for **b** and **c** was shown in (**a**), the persistence

of human NK cells ($hCD45^+hCD56^+$) was shown in (**b**), and the expression of mACE2-CAR ($hCD56^+hLNGFR^+$) was shown in (**c**). **(d)** Representative images of hematoxylin and eosin (H&E) staining for lung tissues of mice after the indicated treatments (4 mice/group). Back arrows indicate A549 tumor nodules. Scale bars, 100 μm.

Supplementary Fig. 5

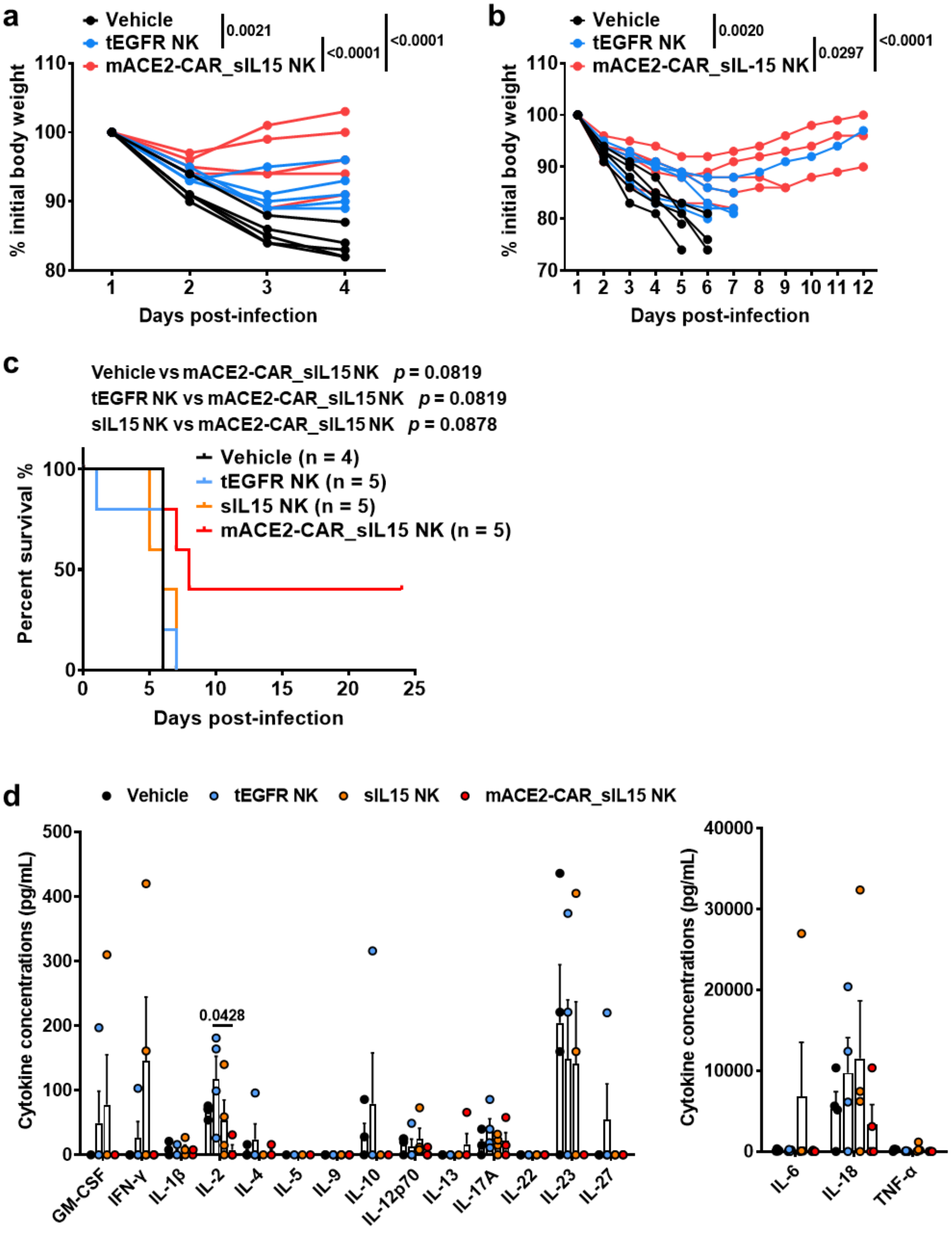

Supplementary Figure 5. (a) K18-hACE2 transgenic mice were infected with 1 × 10^2 plaque-forming units (PFU) of SARS-CoV-2 prior to receiving the treatment with the vehicle (PBS), control tEGFR NK cells, or freeze-thawed mACE2-CAR_sIL15 NK cells. Relative body weights were shown with 5 mice/group. *P* values were determined by two-way ANOVA with repeated measures and adjusted by the Holm-Sidak method at day 4. Data are presented as mean ± SD. **(b)** K18-hACE2 humanized transgenic mice were infected with 1 × 10^3 plaque-forming units (PFU)

of SARS-CoV-2 prior to treatment with the vehicle (PBS), control tEGFR NK cells, or mACE2-CAR_sIL15 NK cells. Relative body weights were shown with 5 mice/group. *P* values were determined by two-way ANOVA with repeated measures and adjusted by the Holm-Sidak method at day 6. Data are presented as mean ± SD. **(c)** K18-hACE2 humanized transgenic mice were infected with 1×10^3 plaque-forming units (PFU) of SARS-CoV-2 prior to treatment with the vehicle (PBS), control tEGFR NK cells, control sIL15 NK cells, or mACE2-CAR_sIL15 NK cells. Survival was summarized with 4 mice in the vehicle group and 5 mice in each of the other groups. *P* values were determined by Kaplan-Meier survival analysis and calculated by the Log-rank test (two-sided). (**d**) Determination of various cytokines in plasma of K18-hACE2 transgenic mice. Mice were infected with 1×10^3 PFU of live SARS-CoV-2 prior to being treated with the vehicle (PBS), control tEGFR NK cells, control sIL15 NK cells, or mACE2-CAR_sIL15 NK cells on the same day. Four days later, mice were sacrificed to collect blood plasma to measure levels of the indicated cytokines by a cytokine release Luminex assay in a BSL3 lab. Data are summarized from 4 different mice in each group. *P* values were determined by one-way ANOVA with multiple comparisons and adjusted by the Holm-Sidak method. Data are presented as mean ± SD.

Supplementary Table 1: Information of antibodies used in flow cytometric assay**.**

**Antibody**	**Conjugation**	**Company**	**Cat#**	**Dilution**
CD56	APC	Beckman Coulter	B46024	1:20
CD56	BV605	BD Bioscience	562780	1:20
CD56	FITC	BD Bioscience	562794	1:20
LNGFR	APC	BD Biosciences	560326	1:20
LNGFR	PE	BD Biosciences	557196	1:20
CD16	FITC	BD Bioscience	555406	1:10
CD69	FITC	BD Bioscience	557049	1:10
CD62L	FITC	BD Bioscience	561914	1:10
KIR-NKAT2	FITC	BD Bioscience	556070	1:10
NKG2A	FITC	Miltenyi Biotec	130-113-563	1:50
NKp44	FITC	Miltenyi Biotec	130-118-542	1:50
CD94	FITC	BD Biosciences	555888	1:10
TRAIL	PE	BD Bioscience	550516	1:50
NKp46	PE	BD Bioscience	557991	1:20
DNAM-1	PE	BD Bioscience	559789	1:10
CD25	PE	BD Bioscience	555432	1:10
NKG2D	PE	BD Bioscience	557940	1:50
CD3	PE	BD Bioscience	555333	1:20
CD3	VioGreen	Miltenyi Biotec	130-113-134	1:50
NKp30	PE	BioLegend	325208	1:20
EGFR	PE	BioLegend	352904	1:50
CD45	BUV395	BD Biosciences	563792	1:20
IFN-γ	BUV395	BD Biosciences	563563	1:20
TNF-α	PE-Cy7	BD Biosciences	557647	1:20
CD107a	FITC	BioLegend	328606	1:200
Anti-His	FITC	ThermoFisher	MA1-81891	1:20
Mouse IgG1	FITC	BD Biosciences	555748	1:10
Mouse IgG1	PE	BD Biosciences	559320	1:10
Mouse IgG1	PE	BioLegend	400111	1:20
goat anti-rabbit IgG	FITC	BD Biosciences	554020	1:20
