# Supplementary material for: Off-the-shelf CAR natural killer cells secreting IL-15 target spike in treating COVID-19
Source: Nat Commun. 2022 May 11;13:2576. doi: 10.1038/s41467-022-30216-8 (PMC9095674; doi:10.1038/s41467-022-30216-8)
Supplement: Supplementary file 2 — Reporting Summary [file 41467_2022_30216_MOESM2_ESM.pdf]

nature portfolio

Corresponding author(s): Michael A. Caligiuri, Jianhua Yu

Last updated by author(s): Mar 24, 2022

Reporting Summary

Nature Portfolio wishes to improve the reproducibility of the work that we publish. This form provides structure for consistency and transparency in reporting. For further information on Nature Portfolio policies, see our Editorial Policies and the Editorial Policy Checklist.

Statistics

For all statistical analyses, confirm that the following items are present in the figure legend, table legend, main text, or Methods section.

n/a	Confirmed	
☐	☒	The exact sample size (*n*) for each experimental group/condition, given as a discrete number and unit of measurement
☐	☒	A statement on whether measurements were taken from distinct samples or whether the same sample was measured repeatedly
☐	☒	The statistical test(s) used AND whether they are one- or two-sided<br>*Only common tests should be described solely by name; describe more complex techniques in the Methods section.*
☒	☐	A description of all covariates tested
☐	☒	A description of any assumptions or corrections, such as tests of normality and adjustment for multiple comparisons
☐	☒	A full description of the statistical parameters including central tendency (e.g. means) or other basic estimates (e.g. regression coefficient) AND variation (e.g. standard deviation) or associated estimates of uncertainty (e.g. confidence intervals)
☐	☒	For null hypothesis testing, the test statistic (e.g. *F*, *t*, *r*) with confidence intervals, effect sizes, degrees of freedom and *P* value noted<br>*Give P values as exact values whenever suitable.*
☒	☐	For Bayesian analysis, information on the choice of priors and Markov chain Monte Carlo settings
☒	☐	For hierarchical and complex designs, identification of the appropriate level for tests and full reporting of outcomes
☒	☐	Estimates of effect sizes (e.g. Cohen's *d*, Pearson's *r*), indicating how they were calculated

Our web collection on statistics for biologists contains articles on many of the points above.

Software and code

Policy information about availability of computer code

Data collection	Aura Version 3.2 was used to collected Bioluminescent imagings; BD FACSDiva version 6 was used to collect data for flow cytometry; RTCA eSight Software 1.0.3 was used to collect data for real-time cell analysis (RTCA).
Data analysis	Statistical software: GraphPad Prism 9; Flow cytometry data analysis software: FlowJo_v10. Bioluminescent imaging analysis software: Aura Version 3.2; RTCA analysis software: RTCA eSight Software 1.0.3.

For manuscripts utilizing custom algorithms or software that are central to the research but not yet described in published literature, software must be made available to editors and reviewers. We strongly encourage code deposition in a community repository (e.g. GitHub). See the Nature Portfolio guidelines for submitting code & software for further information.

Data

Policy information about availability of data

All manuscripts must include a data availability statement. This statement should provide the following information, where applicable:

- Accession codes, unique identifiers, or web links for publicly available datasets
- A description of any restrictions on data availability
- For clinical datasets or third party data, please ensure that the statement adheres to our policy

The authors declare that all data supporting the findings of this study are available within the paper and its supplementary information files.

Field-specific reporting

Please select the one below that is the best fit for your research. If you are not sure, read the appropriate sections before making your selection.

☒ Life sciences ☐ Behavioural & social sciences ☐ Ecological, evolutionary & environmental sciences

For a reference copy of the document with all sections, see nature.com/documents/nr-reporting-summary-flat.pdf

Life sciences study design

All studies must disclose on these points even when the disclosure is negative.

Sample size	No statistical method was used to determine the sample size. A reasonable sample sizes were determined based on our and other investigators experience and similar research reported in the literature to ensure adequate reproducibility of results. The sample size and associated statistics are indicated in the figures and respective legends.
Data exclusions	No data were excluded from the analyses.
Replication	All experiments were performed with sufficient biological and technical replicates with cells and animals per group in order to demonstrate statistical significance. Number of replicates in each experiment is indicated in the corresponding figure or legend.
Randomization	For umbilical cord blood units used to isolate NK cells and generate engineered NK cells were randomly picked up. For in vivo experiments, NSG mice were randomly divided into respective groups for experiments after tumor establishment. K18-hACE2 mice were divided into different into groups based on cages due to the P3 Lab's specific experimental condition.
Blinding	Experiments blinding was not necessary during the experimentation and data collection. As the results were quantitative and did not require critical interpretations or subjective judgment. Also these groups were well defined and studied using standard protocols.

Reporting for specific materials, systems and methods

We require information from authors about some types of materials, experimental systems and methods used in many studies. Here, indicate whether each material, system or method listed is relevant to your study. If you are not sure if a list item applies to your research, read the appropriate section before selecting a response.

Materials & experimental systems

n/a	Involved in the study	
☐	☒	Antibodies
☐	☒	Eukaryotic cell lines
☒	☐	Palaeontology and archaeology
☐	☒	Animals and other organisms
☐	☒	Human research participants
☒	☐	Clinical data
☒	☐	Dual use research of concern

Methods

n/a	Involved in the study	
☒	☐	ChIP-seq
☐	☒	Flow cytometry
☒	☐	MRI-based neuroimaging

Antibodies

Antibodies used

APC mouse anti-human CD56, Clone: N901, Beckman Coulter, Cat# IM2474U;
BV605 mouse anti-human CD56, Clone: NCAM16.2, BD Biosciences, Cat# 562780;
FITC mouse anti-human CD56, Clone: B159, BD Biosciences, Cat# 562794;
AF647 mouse anti-human LNGFR, Clone: C40-1457, BD Biosciences, Cat# 560326;
PE mouse anti-human LNGFR, Clone: C40-1457, BD Biosciences, Cat# 557196;
FITC mouse anti-human CD16, Clone: 3G8, BD Biosciences, Cat# 555406;
FITC mouse anti-human CD69, Clone: FN50, BD Biosciences, Cat# 557049;
FITC mouse anti-human KIR-NKAT2, Clone: DX27, BD Biosciences, Cat# 556070;
FITC mouse anti-human CD62L, Clone: DREG-56, BD Biosciences, Cat# 561914;
FITC mouse anti-human NKG2A, Clone: REA110, Miltenyi Biotec, Cat# 130-113-563;
FITC mouse anti-human NKp44, Clone: 2.29, Miltenyi Biotec, Cat# 130-118-542;
FITC mouse anti-human CD94, Clone: HP-3D9, BD Biosciences, Cat# 555888;
PE mouse anti-human TRAIL, Clone: RIK-2, BD Biosciences, Cat# 550516;
PE mouse anti-human NKp46, Clone: 9E2, BD Biosciences, Cat# 557991;
PE mouse anti-human DNAM-1, Clone: DX11, BD Biosciences, Cat# 559789;
PE mouse anti-human CD25, Clone: M-A251, BD Biosciences, Cat# 555432;
PE mouse anti-human NKG2D, Clone: 1D11, BD Biosciences, Cat# 557940;
PE mouse anti-human CD3, Clone: UCHT1, BD Biosciences, Cat# 555333;
VioGreen mouse anti-human CD3, Clone: BW264/56, Miltenyi Biotec, Cat# 130-113-134;

PE mouse anti-human NKp30, Clone: P30-15, BioLegend, Cat# 325208;
PE mouse anti-human EGFR, Clone: AY13, BioLegend, Cat# 352904;
BUV395 mouse anti-human CD45, Clone: HI30, BD Biosciences, Cat# 563792;
BUV395 mouse anti-human IFN-γ, Clone: B27, BD Biosciences, Cat# 563563;
PE-Cy7 mouse anti-human TNF-α, Clone: MAb11, BD Biosciences, Cat# 557647;
FITC mouse anti-human CD107a, Clone: H4A3, BioLegend, Cat# 328606;
FITC anti-His, Clone: AD1.1.10, ThermoFisher, Cat# MA1-81891;
FITC goat anti-rabbit IgG, BD Biosciences, Cat# 554020;
FITC Mouse IgG1, Clone: MOPC-21, BD Biosciences, Cat# 555748;
PE Mouse IgG1, Clone: MOPC-21, BD Biosciences, Cat# 559320;
PE Mouse IgG1, Clone: MOPC-21, BioLegend, Cat# 400111;
SARS-CoV-2 (2019-nCoV) Spike S1 Antibody, Rabbit mAb, Sino Biological, Cat# 40150-R007;
InVivoMAb anti-mouse NK1.1, Clone: PK136, Bio X cell, Cat# BE0036;
InVivoMAb anti-mouse CD8α, Clone: 2.43, Bio X cell, Cat# BE0061;
InVivoMAb anti-mouse CD4, Clone: GK1.5, Bio X cell, Cat# BE0003-1.

Validation

All antibodies are commercially available flow cytometry antibodies for staining human samples and validated by the manufacturer.

APC mouse anti-human CD56, Clone: N901, Beckman Coulter, Cat# IM2474U:
Please see the manufacturer's website link for application. https://www.beckman.com/reagents/coulter-flow-cytometry/antibodies-and-kits/single-color-antibodies/cd56

BV605 mouse anti-human CD56, Clone: NCAM16.2, BD Biosciences, Cat# 562780:
Please see the manufacturer's website link for application. https://www.bdbiosciences.com/en-us/products/reagents/flow-cytometry-reagents/research-reagents/single-color-antibodies-ruo/bv605-mouse-anti-human-cd56.562780

FITC mouse anti-human CD56, Clone: B159, BD Biosciences, Cat# 562794:
Please see the manufacturer's website link for application. https://www.bdbiosciences.com/en-us/products/reagents/flow-cytometry-reagents/research-reagents/single-color-antibodies-ruo/fitc-mouse-anti-human-cd56-ncam-1.562794

AF647 mouse anti-human LNGFR, Clone: C40-1457, BD Biosciences, Cat# 557196:
Please see the manufacturer's website link for application. https://www.bdbiosciences.com/en-us/products/reagents/flow-cytometry-reagents/research-reagents/single-color-antibodies-ruo/alexa-fluor-647-mouse-anti-human-cd271.560326

PE mouse anti-human LNGFR, Clone: C40-1457, BD Biosciences, Cat# 557196:
Please see the manufacturer's website link for application. https://www.bdbiosciences.com/en-us/products/reagents/flow-cytometry-reagents/research-reagents/single-color-antibodies-ruo/pe-mouse-anti-human-cd271.557196

FITC mouse anti-human CD16, Clone: 3G8, BD Biosciences, Cat# 555406:
Please see the manufacturer's website link for application. https://www.bdbiosciences.com/en-us/products/reagents/flow-cytometry-reagents/research-reagents/single-color-antibodies-ruo/fitc-mouse-anti-human-cd16.555406

FITC mouse anti-human CD69, Clone: FN50, BD Biosciences, Cat# 557049:
Please see the manufacturer's website link for application. https://www.bdbiosciences.com/en-us/products/reagents/flow-cytometry-reagents/research-reagents/single-color-antibodies-ruo/fitc-mouse-anti-human-cd69.557049

FITC mouse anti-human KIR-NKAT2, Clone: DX27, BD Biosciences, Cat# 556070:
Please see the manufacturer's website link for application. https://www.bdbiosciences.com/en-us/products/reagents/flow-cytometry-reagents/research-reagents/single-color-antibodies-ruo/fitc-mouse-anti-human-cd158b1-b2-j.556070

FITC mouse anti-human CD62L, Clone: DREG-56, BD Biosciences, Cat# 561914:
Please see the manufacturer's website link for application. https://www.bdbiosciences.com/en-us/products/reagents/flow-cytometry-reagents/research-reagents/single-color-antibodies-ruo/fitc-mouse-anti-human-cd62l.561914

FITC mouse anti-human NKG2A, Clone: REA110, Miltenyi Biotec, Cat# 130-113-563:
Please see the manufacturer's website link for application. https://www.miltenyibiotec.com/US-en/products/cd159a-nkg2a-antibody-anti-human-reafinity-rea110.html#gref

FITC mouse anti-human NKp44, Clone: 2.29, Miltenyi Biotec, Cat# 130-118-542:
Please see the manufacturer's website link for application.https://www.miltenyibiotec.com/US-en/products/cd336-nkp44-antibody-anti-human-2-29.html#vio-bright-fitc:100-tests-in-200-ul

FITC mouse anti-human CD94, Clone:HP-3D9, BD Biosciences, Cat# 555888:
Please see the manufacturer's website link for application. https://www.bdbiosciences.com/en-us/products/reagents/flow-cytometry-reagents/research-reagents/single-color-antibodies-ruo/fitc-mouse-anti-human-cd94.555888

PE mouse anti-human TRAIL, Clone: RIK-2, BD Biosciences, Cat# 550516:
Please see the manufacturer's website link for application. https://www.bdbiosciences.com/en-us/products/reagents/flow-cytometry-reagents/research-reagents/single-color-antibodies-ruo/pe-mouse-anti-human-cd253.550516

PE mouse anti-human NKp46, Clone: 9-E2, BD Biosciences, Cat# 557991:
Please see the manufacturer's website link for application. https://www.bdbiosciences.com/en-us/products/reagents/flow-cytometry-reagents/research-reagents/single-color-antibodies-ruo/pe-mouse-anti-human-cd335-nkp46.557991

PE mouse anti-human DNAM-1, Clone: DX11, BD Biosciences, Cat# 559789:

Please see the manufacturer's website link for application. https://www.bdbiosciences.com/en-us/products/reagents/flow-cytometry-reagents/research-reagents/single-color-antibodies-ruo/pe-mouse-anti-human-cd226.559789

PE mouse anti-human CD25, Clone: M-251, BD Biosciences, Cat# 555432:
Please see the manufacturer's website link for application. https://www.bdbiosciences.com/en-us/products/reagents/flow-cytometry-reagents/research-reagents/single-color-antibodies-ruo/pe-mouse-anti-human-cd25.555432

PE mouse anti-human NKG2D, Clone: 1D11, BD Biosciences, Cat# 557940;
Please see the manufacturer's website link for application. https://www.bdbiosciences.com/en-us/products/reagents/flow-cytometry-reagents/research-reagents/single-color-antibodies-ruo/pe-mouse-anti-human-cd314-nkg2d.557940

PE mouse anti-human CD3, Clone: UCHT1, BD Biosciences, Cat# 555333;
Please see the manufacturer's website link for application. https://www.bdbiosciences.com/en-us/products/reagents/flow-cytometry-reagents/research-reagents/single-color-antibodies-ruo/pe-mouse-anti-human-cd3.555333

VioGreen mouse anti-human CD3, Clone: BW264/56, Miltenyi Biotec, Cat# 130-113-134:
Please see the manufacturer's website link for application. https://www.miltenyibiotec.com/US-en/products/cd3-antibody-anti-human-bw264-56.html#viogreen:100-tests-in-200-ul

PE mouse anti-human NKp30, Clone: P30-15, BioLegend, Cat# 325208;
Please see the manufacturer's website link for application. https://www.biolegend.com/en-us/search-results/pe-anti-human-cd337-nkp30-antibody-3855

PE mouse anti-human EGFR, Clone: AY13, BioLegend, Cat# 352904;
Please see the manufacturer's website link for application. https://www.biolegend.com/en-us/products/pe-anti-human-egfr-antibody-7432

BUV395 mouse anti-human CD45, Clone: HI30, BD Biosciences, Cat# 563792:
Please see the manufacturer's website link for application. https://www.bdbiosciences.com/en-us/products/reagents/flow-cytometry-reagents/research-reagents/single-color-antibodies-ruo/buv395-mouse-anti-human-cd45.563792

BUV395 mouse anti-human IFN-γ, Clone: B27, BD Biosciences, Cat# 563563:
Please see the manufacturer's website link for application. https://www.bdbiosciences.com/en-us/products/reagents/flow-cytometry-reagents/research-reagents/single-color-antibodies-ruo/buv395-mouse-anti-human-ifn.563563

PE-Cy7 mouse anti-human TNF-α, Clone: MAb11, BD Biosciences, Cat# 557647:
Please see the manufacturer's website link for application. https://www.bdbiosciences.com/en-us/products/reagents/flow-cytometry-reagents/research-reagents/single-color-antibodies-ruo/pe-cy-7-mouse-anti-human-tnf.557647

FITC mouse anti-human CD107a, Clone: H4A3, BioLegend, Cat# 328606:
Please see the manufacturer's website link for application. https://www.biolegend.com/en-us/products/fitc-anti-human-cd107a-lamp-1-antibody-4966

FITC anti-His, Clone: AD1.1.10, ThermoFisher, Cat# MA1-81891:
Please see the manufacturer's website link for application. https://www.thermofisher.com/antibody/product/6x-His-Tag-Antibody-clone-AD1-1-10-Monoclonal/MA1-81891

FITC goat anti-rabbit IgG, BD Biosciences, Cat# 554020:
Please see the manufacturer's website link for application. https://www.bdbiosciences.com/en-us/products/reagents/flow-cytometry-reagents/research-reagents/single-color-antibodies-ruo/fitc-goat-anti-rabbit-igg.554020

FITC Mouse IgG1, Clone: MOPC-21, BD Biosciences, Cat# 555748:
Please see the manufacturer's website link for application. https://www.bdbiosciences.com/en-us/products/reagents/flow-cytometry-reagents/flow-cytometry-controls-and-lysates/fitc-mouse-igg1-isotype-control.555748

PE Mouse IgG1, Clone: MOPC-21, BD Biosciences, Cat# 559320:
Please see the manufacturer's website link for application. https://www.bdbiosciences.com/en-us/products/reagents/flow-cytometry-reagents/flow-cytometry-controls-and-lysates/pe-mouse-igg1-isotype-control.559320

PE Mouse IgG1, Clone: MOPC-21, BioLegend, Cat# 400111:
Please see the manufacturer's website link for application. https://www.biolegend.com/en-us/products/pe-mouse-igg1-kappa-isotype-ctrl-1408

SARS-CoV-2 (2019-nCoV) Spike S1 Antibody, Rabbit mAb, Sino Biological, Cat# 40150-R007
Please see the manufacturer's website link for application. https://www.sinobiological.com/antibodies/cov-spike-40150-r007?gclid=Cj0KCQjwuMuRBhCJARIsAHXdnqOUN-XE3eygJgtDQr2oE7m_Zfrwc0xOFbYD5JptWwL4IE6AAJ5BbMMaArELEALw_wcB

InVivoMAb anti-mouse NK1.1, Clone: PK136, Bio X cell, Cat# BE0036
Please see the manufacturer's website link for application. https://bxcell.com/product/nk-1-1/

InVivoMAb anti-mouse CD8α, Clone: 2.43, Bio X cell, Cat# BE0061
Please see the manufacturer's website link for application. https://bxcell.com/product/m-cd8a-2/

InVivoMAb anti-mouse CD4, Clone: GK1.5, Bio X cell, Cat# BE0003-1

Please see the manufacturer's website link for application. https://bxcell.com/product/m-cd4/

Eukaryotic cell lines

Policy information about cell lines

Cell line source(s)	The A549 cell line was purchased from the American Type Culture Collection (ATCC); The GP2-293 packaging cell line was purchased from Takara Bio;
Authentication	None of the cell lines used were authenticated.
Mycoplasma contamination	All cell lines were tested negative for mycoplasma contamination.
Commonly misidentified lines (See ICLAC register)	No commonly misidentified cell lines were used in this study.

Animals and other organisms

Policy information about studies involving animals; ARRIVE guidelines recommended for reporting animal research

Laboratory animals	Female NSG mice (8-12 weeks old) were housed in City of Hope Animal Facility with light cycle (14-hour light/10-hour dark cycle or 12 light/12 dark cycle) and temperatures of 65-75°F with 40-60% humidity. Heterozygous K18-hACE c57BL/6J mice (2B6.Cg-Tg(K18-ACE2)2Prlmn/J) (6-8 weeks old) were housed in an animal biosafety level 3 (ABSL3) facility (14-hour light/10-hour dark cycle or 12 light/12 dark cycle) with temperatures of 65-75°F with 40-60% humidity.
Wild animals	The study did not involve wild animals.
Field-collected samples	The study did not involve samples collected from the field.
Ethics oversight	The experiments of NSG mice were approved by the City of Hope Animal Care and the experiments of K18-hACE2 mice were approved by the institutional animal care and committee of Northern Arizona University.

Note that full information on the approval of the study protocol must also be provided in the manuscript.

Human research participants

Policy information about studies involving human research participants

Population characteristics	Umbilical cord blood units were obtained from StemCyte Inc., and the covariate-relevant population characteristics were not revealed.
Recruitment	Participants were recruited randomly by StemCyte Inc., and we did not outline any self-selection bias.
Ethics oversight	All participates who provided informed consent had samples collected under a protocol approved by the Institution Review Board at City of Hope National Medical Center.

Note that full information on the approval of the study protocol must also be provided in the manuscript.

Flow Cytometry

Plots

Confirm that:

☒ The axis labels state the marker and fluorochrome used (e.g. CD4-FITC).

☒ The axis scales are clearly visible. Include numbers along axes only for bottom left plot of group (a 'group' is an analysis of identical markers).

☒ All plots are contour plots with outliers or pseudocolor plots.

☒ A numerical value for number of cells or percentage (with statistics) is provided.

Methodology

Sample preparation	For the isolation of lymphocytes from murine spleen, liver and lung, a single cell suspension was generated by mashing the organ through a cell strainer.<br>Cells were washed in FACS buffer (PBS + 1% FBS) before labeling with flurorochrome-conjugated antibodies. Labeling of single-cell suspensions was performed at room temperature for 20 min avoiding the light.<br>For intracellular cytokine stainings were carried out upon permeabilization and fixation with BD fix/perm Kit.
Instrument	Fortessa X20 (BD Biosciences) was used to collect data of flow cytometry.

Software	BD FACSDiva™ Software was used to collect data and FlowJo_v10 was used to analyzed the data for flow cytometry assay.
Cell population abundance	The purity of sorted cells was detected via BD FACSAriaTM Fusion and samples with purity > 95% were used.
Gating strategy	Doublets (based on FSC and SSC values) and dead cells (based on live/dead staining) were gated out from the starting cell population to select only live, single cells.<br>In human NK cells, LNGFR expression, surface markers (e.g. CD16, CD94 et al.) expression, CD107a expression, IFN-γ expression, TNF-α expression were gated from NK cells which were defined as CD56+ population;<br>In mice samples, human NK cells were defined as CD56+CD45+ population, and the expression of LNGFR was gated in human NK cells. The boundary between positive and negative cell population was defined using isotype controls and unstained controls.

☒ Tick this box to confirm that a figure exemplifying the gating strategy is provided in the Supplementary Information.
